# Comparative genome-wide analysis of WRKY, MADS-box and MYB transcription factor families in *Arabidopsis* and rice

**DOI:** 10.1038/s41598-021-99206-y

**Published:** 2021-10-04

**Authors:** Muhammad-Redha Abdullah-Zawawi, Nur-Farhana Ahmad-Nizammuddin, Nisha Govender, Sarahani Harun, Norfarhan Mohd-Assaad, Zeti-Azura Mohamed-Hussein

**Affiliations:** 1 Institute of Systems Biology (INBIOSIS), Universiti Kebangsaan Malaysia, 43600 UKM, Bangi, Selangor Malaysia; 2 Department of Applied Physics, Faculty of Science and Technology, Universiti Kebangsaan Malaysia, 43600 UKM, Bangi, Selangor Malaysia

**Keywords:** Data mining, Data processing, Gene ontology, Gene regulatory networks, Genome informatics, Phylogeny

## Abstract

Transcription factors (TFs) form the major class of regulatory genes and play key roles in multiple plant stress responses. In most eukaryotic plants, transcription factor (TF) families (WRKY, MADS-box and MYB) activate unique cellular-level abiotic and biotic stress-responsive strategies, which are considered as key determinants for defense and developmental processes. *Arabidopsis* and rice are two important representative model systems for dicot and monocot plants, respectively. A comprehensive comparative study on 101 *OsWRKY*, 34 *OsMADS box* and 122 *OsMYB* genes (rice genome) and, 71 *AtWRKY*, 66 *AtMADS box* and 144 *AtMYB* genes (*Arabidopsis* genome) showed various relationships among TFs across species. The phylogenetic analysis clustered WRKY, MADS-box and MYB TF family members into 10, 7 and 14 clades, respectively. All clades in WRKY and MYB TF families and almost half of the total number of clades in the MADS-box TF family are shared between both species. Chromosomal and gene structure analysis showed that the *Arabidopsis*-rice orthologous TF gene pairs were unevenly localized within their chromosomes whilst the distribution of exon–intron gene structure and motif conservation indicated plausible functional similarity in both species. The abiotic and biotic stress-responsive *cis*-regulatory element type and distribution patterns in the promoter regions of *Arabidopsis* and rice WRKY, MADS-box and MYB orthologous gene pairs provide better knowledge on their role as conserved regulators in both species. Co-expression network analysis showed the correlation between WRKY, MADs-box and MYB genes in each independent rice and Arabidopsis network indicating their role in stress responsiveness and developmental processes.

## Introduction

Transcription factors (TFs) are characterized as proteins with at least one domain that corresponds to a specific-DNA binding site and control the transcriptional regulatory schemes in plant cells. TFs regulate the spatio-temporal expression of target genes involved in plant growth and development, and response systems to the terrestrial environment. TF mediated responses are established upon intrinsic and external signals in controlling and coordinating the activation or repression of functional gene expression^1–4^. TFs have a unique DNA binding site, known as the cis-regulatory element (CREs) in the promoter region of a gene for independent regulation, induction and/or cross-regulatory activation such as epigenetics and signalling process. TFs are categorized according to the conserved motifs in DNA-binding domains (DBDs) such as NAC, SBP, MADS-box, WRKY, B3 among others. In plants, the distribution of TF families is assumed plant species-specific. Currently, 58 different TF families are deposited in the PlantTFDB database and they have been exclusively characterized in model plants^3^. Amongst these TF families, WRKY, MADS-box and MYB are the most important transcriptional regulators that are widely distributed in the plant kingdom and actively involved in plant development and, biotic and abiotic stress responses^4^.

The WRKY, the seventh-largest family of TFs is involved in the developmental processes and defense responses such as seed germination, pollen development, hormonal regulation, biosynthesis of secondary metabolites^5^. WRKY TF family is characterized by a WRKY signature domain that contains WD containing amino acid residues positioned at the N-terminus and a zinc-finger domain at the C-terminus of the sequence. It consists of approximately 60–70 amino acid residues with WRKYGQK /WRKYGKK motif for DNA-binding promoter element or W-Box (TTGACC/T) recognition^6,7^. In the MYB family, TFs are involved in plant development and defense responses including cell cycle, cell morphogenesis, central circadian oscillator and regulation of stress signalling^8,9^. The MYB domain contains three irregular repeats that form a helix-turn-helix (HTH) structure of about 53 amino acids^10^. In MYB proteins, the R1, R2, R3 (conventional) and R4 groups (numbered according to the number of the adjacent repeats) of MYB-domain repeats stabilize the DNA-binding structure^11^. The TFs with MCM1/AGAMOUS/DEFICIENS/SRF (MADS)-box regulate the developmental processes such as seed germination, vegetative growth, the transition from vegetative to reproductive growth, floral development and senescence and regulating the abiotic and biotic stress tolerance. They contain a conserved MADS domain consists of 60-amino acid long at the N-terminal and recognizes the CArG-box DNA motif (CC[A/]_6_GG) in the target genes. Generally, they are classified into two lineages namely, type I and type II. Type 1 contains MADS domain and an extended highly variable carboxy-terminal domain whilst type II contains four conserved domains known as the MIKC that consists of M-domain, Intervening-domain, Keratin-like domain and the carboxy-terminal domain^12^.

Rice and *Arabidopsis* are important non-halophytes model plants for monocot and dicot crops, respectively. They are short-rotation plants with high sensitivity to stressors; oxidative, osmotic and ion/salt stress^13,14^. The first rice genome was published in 2006 and has become an excellent model system for the economically important related monocotyledons crops such as maize, wheat, sorghum and barley. On the other hand, the dicotyledonous *A. thaliana* was the first model plant with a completed genome sequence published in the year 2000 (http://www.arabidopsis.org)^13^. It has been actively used by the plant research community in revolutionizing genetics and breeding studies^14^. More than 5% of the *Arabidopsis* genes encode for TFs and only about 7% of them have been functionally and genetically characterized. The genome size of *Arabidopsis* is approximately 135 megabase pairs, about one-fourth of the size of the rice genome and contains up to 30 000 genes. Currently, there are 2296 and 2408 genes encoding TFs in *Arabidopsis* and rice, respectively^15^.

The *Arabidopsis* and rice WRKY, MADS-box and MYB TF families are reported to show diverse functional roles. In rice, the OsMYB-R1 gene regulates multiple stress tolerance^16^, RADIALS-LIKE3 (OsRL3) promotes dark-induced leaf senescence and reduce susceptibility to salt stress^17^, OsWRKY74 and OsWRKY28 regulate the phosphate homeostasis^18,19^ and OsMADS27 regulates root development under a salt-tolerant condition^20^. In *Arabidopsis*, AGL21, the MADS-box TF acts as environmental surveillance during seed germination. There are 109 and 74 WRKY families in rice and *Arabidopsis*, respectively^21^. The MYB TF family with up to180 members is the largest TF family in *Arabidopsis* and rice^9^. The MADS-box TF family contains more than 100 members and are generally involved in almost every developmental process of a higher plant^22^.

TFs are an important component in complex regulatory networks established by plants during their response to stressors^19–21^. They either enhance or suppress the expression of genes that are directly associated with target resistance genes. In this study, the WRKY, MADS-box and MYB TF families from rice and *Arabidopsis* were identified and collated for a comprehensive in silico genome-wide analysis in the search for conserved functional roles between different TF families and species. The phylogenetic relationship of the exon–intron arrangement, conserved motif analysis, and promoter analysis of stress-responsive *cis*-regulatory elements present in the orthologous gene pairs (*Arabidopsis* and rice) of three WRKY, MADS-box and MYB TF families are investigated to provide useful insights on the conserved regulatory modules of TFs with potential manipulation for plant biotechnology and breeding programmes.

## Materials and methods

### Data resources

Genes of *Arabidopsis thaliana* and *Oryza sativa* WRKY, MADS-box and MYB encoding transcription factors (TFs) were retrieved from Plant Transcription Factor Database v5.0 (PlantTFDB 5.0; http://planttfdb.cbi.pku.edu.cn)^15^. The corresponding protein-coding sequences were obtained from Phytozome 12.1 (https://phytozome.jgi.doe.gov/pz/portal.html)^23^.

### Multiple sequence alignment and phylogenetic analysis

The multiple sequence alignment (MSA) was conducted using ClustalW v2.1 software with the following parameters set: open penalty of 10 gaps and gap extension at 0.1 to 0.2^24^ followed by the phylogenetic tree construction using MEGA v7.2 software with the Neighbor-Joining (NJ) method with 1000 bootstrap replicates^25,26^. The phylogenetic tree was visualized and annotated using FigTree software v1.4.4 (http://tree.bio.ed.ac.uk/software/figtree/)^27^.

### Chromosomal location analysis

The chromosomal location analysis of the WRKY, MADS-box and MYB TF gene families were performed using TAIR Chromosome Map Tool (https://www.arabidopsis.org/jsp/ChromosomeMap/tool.jsp)^28^ for *Arabidopsis* and Oryzabase Chromosome Map Tool (http://viewer.shigen.info/oryzavw/maptool/MapTool.do) for rice^29^. Genes separated by less than five gene loci at 100 kb distance were considered as tandem duplicates^30^.

### Exon–intron arrangement and motifs search distributions

The exon–intron structural features of WRKY, MADS-box and MYB TF genes were visualized using Gene Structure Display Server 2.0 (http://gsds.cbi.pku.edu.cn/)^31^. The conserved motifs of the target sequences were identified by Multiple Expectation Maximization for Motif Elicitation (MEME) Suite Software (http://meme-suite.org/) using the following parameters: maximum number motifs is set at 20 and allow zero or one occurrence per sequence (zoops) mode^32^. Pfam online tool (https://pfam.xfam.org) was employed for conserved motif annotation^33^.

### Prediction of *cis*-regulatory element on promoter regions

Promoter region and the *cis-*regulatory elements (CREs) of the WRKY, MADS-box and MYB target sequences were examined using a web-based tool, the PLANTCARE (http://bioinformatics.psb.ugent.be/webtools/plantcare/html)^34^ followed by the visualization of CREs using Illustrator for Biological Sequences (IBS) software (http://ibs.biocuckoo.org)^35^.

### In silico co-expression analysis and functional similarity between orthologous gene pair

Gene identifier of orthologous pair for WRKY, MADS-box and MYB target sequences was searched against PLANT co-expression database (PLANEX, http://planex.plantbioinformatics.org)^36^. The co-expression data were retrieved, and the networks were visualized using Cytoscape v3.7.0 software^37^. Functional similarity of the co-expression network was measured using kappa value from PLANEX database^36^ that represents the distance of co-expression data between rice and *Arabidopsis*.

## Results

### Phylogenetic analysis of WRKY, MADS-box and MYB genes in *Arabidopsis* and rice

**Figure 1:**
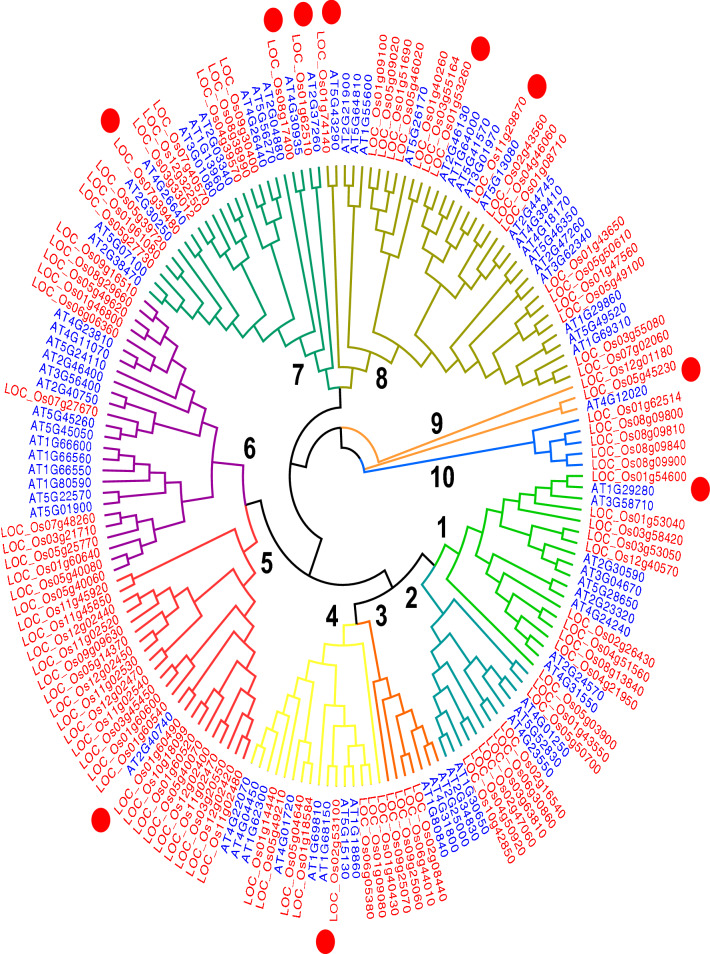
Phylogenetic tree of collated rice and *Arabidopsis* full-length WRKY protein sequences. Red dots represent the rice-Arabidopsis orthologous gene pairs. The tree is built using the neighbor-joining (NJ) method (MEGA7.0 software) and are divided into ten clades, numbered in bold.

**Figure 2:**
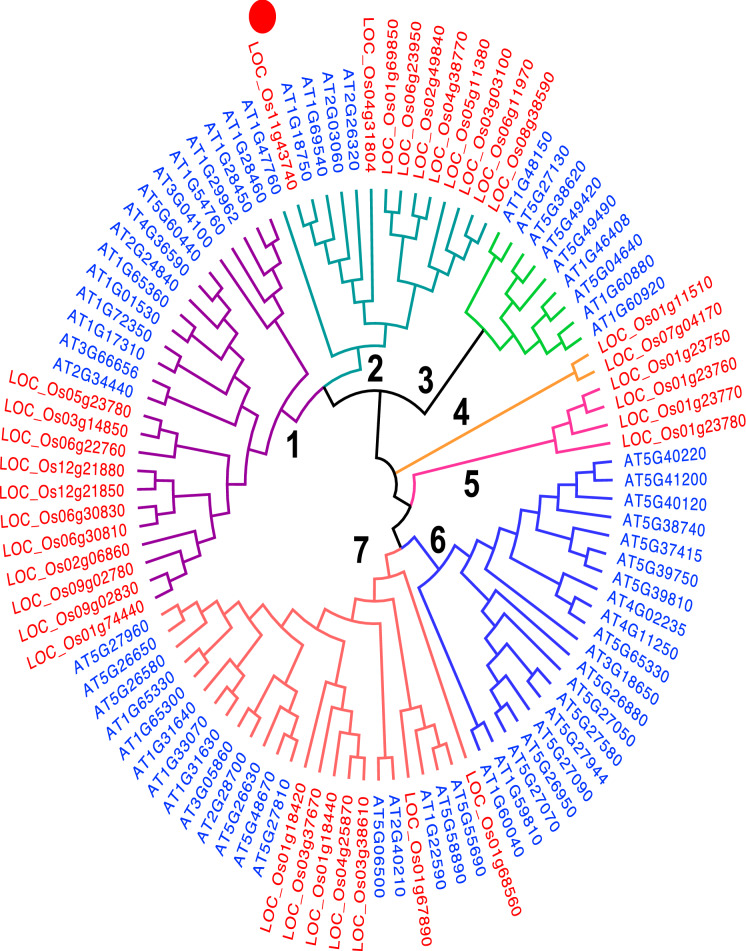
Phylogenetic tree of collated rice and *Arabidopsis* full-length MADS-box protein sequences. Red dots represent the rice-Arabidopsis orthologous gene pairs. The tree is built using the neighbor-joining (NJ) method (MEGA7.0 software) and are divided into seven clades, numbered in bold.

**Figure 3:**
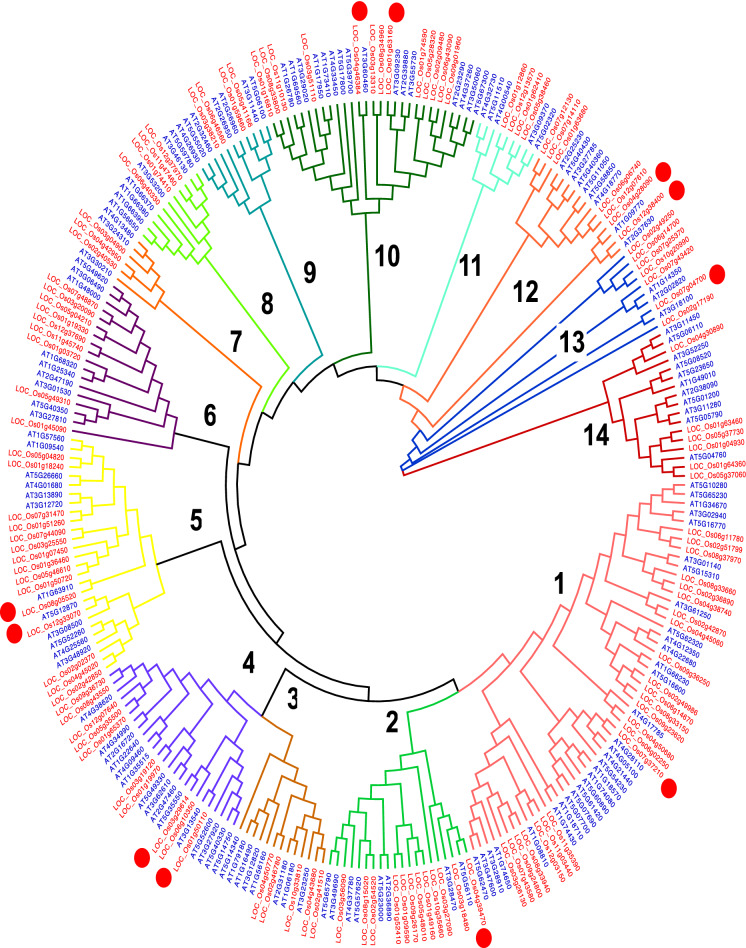
Phylogenetic tree of collated rice and *Arabidopsis* full-length MYB protein sequences. Red dots represent the rice-Arabidopsis orthologous gene pairs. The tree is built using the neighbor-joining (NJ) method (MEGA7.0 software) and are divided into 14 clades, numbered in bold.

101 *OsWRKY*, 34 *OsMADS box* and 122 *OsMYB* sequences were identified in rice and 72 *AtWRKY*, 66 *AtMADS box* and 144 *AtMYB* sequences were identified in *Arabidopsis* after the repetitive and redundant gene sequences were removed. A phylogenetic tree for the WRKY transcription factor (TF) family was built from 173 collated *Arabidopsis* and rice WRKY genes. 101 *OsWRKY* and 72 *AtWRKY* genes are distributed in all clades except clade 5 where only one *Arabidopsis* gene (*AtWRKY*) is present among 22 rice genes (*OsWRKY*) whilst Clade 10 contains WRKY genes from rice only. The highest gene number (GN) is observed in clade 8 (GN = 37), followed by clade 6 (GN = 26), clade 7 (GN = 24) and clade 5 (GN = 23). Clade 9 is the smallest with a GN = 3 (Fig. 1). A phylogenetic tree of the MADS-box TF family constructed from 66 *AtMADS-box* and 34 *OsMADS-box* genes shows consistent distribution among 14 clades. Clade 1 and clade 7 are the biggest clusters with a similar size (GN = 25), followed by clade 6 (GN = 20), clade 2 (GN = 15), clade (GN = 9) and clade 5 (GN = 5). Clade 4 is the smallest with GN = 2. Clade 3 and clade 6 contain gene members from *AtMADS-box* only while clade 4 and clade 5 are unique to *OsMADS-box* members (Fig. 2). A phylogenetic tree of the MYB TF family shows 14 clades, with fairly even Arabidopsis and rice genes representation. Clade 1 is the biggest cluster (GN = 54), followed by clade 10 (GN = 29), clade 7 (GN = 27), clade 4 (GN = 25) and clade 7 (GN = 6) (Fig. 3). In each TF family phylogenetic tree, the orthologous gene pairs identified by red circles were selected for subsequent analysis. A total of 22 orthologous gene pairs are obtained as following: WRKY;10, MADS-box; 1 and MYB; 11 (Figs. 1, 2, 3).

### Distribution of the WRKY, MADS-box and MYB orthologous genes in *Arabidopsis* and *Oryza sativa* chromosomes

**Figure 4:**
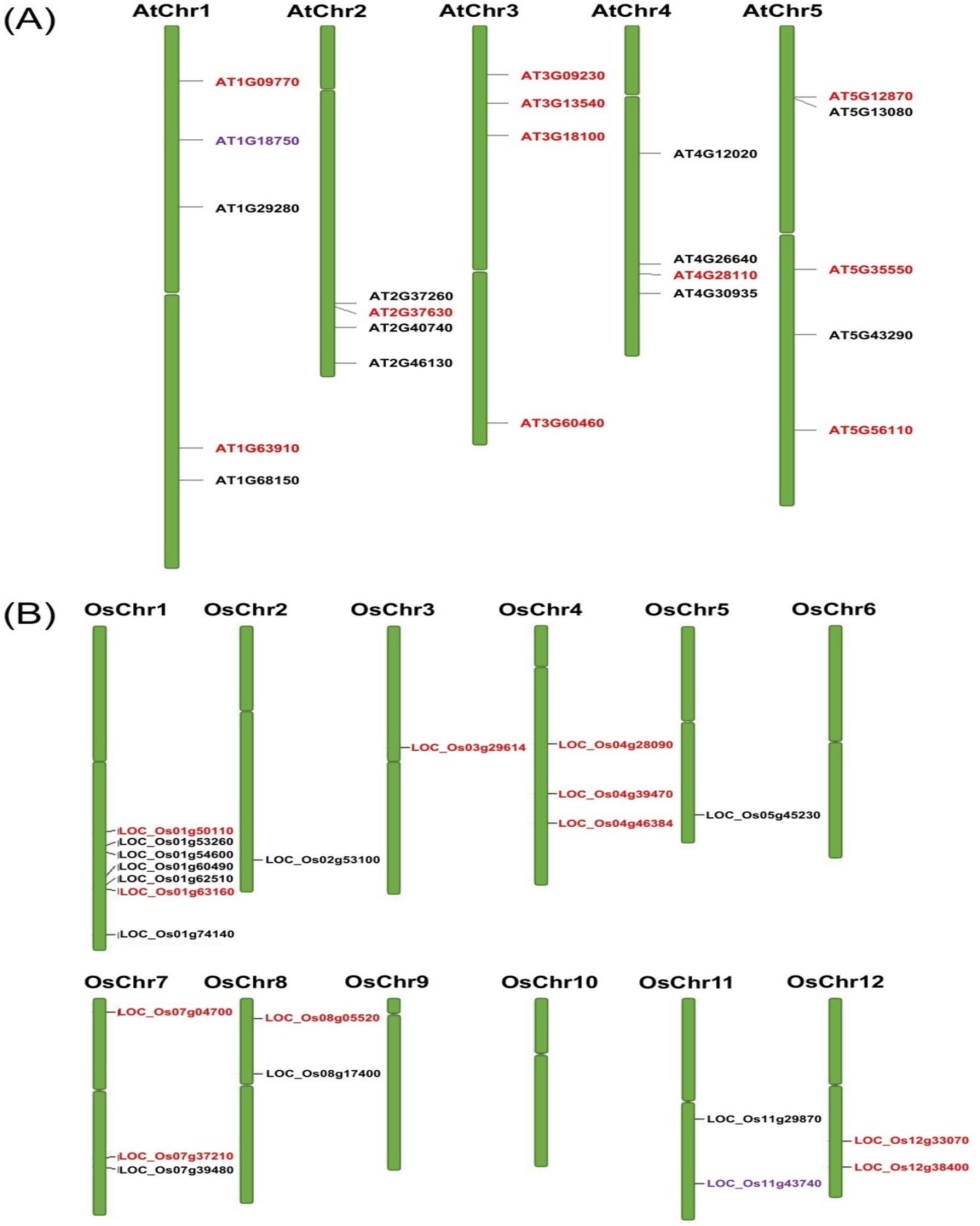
The chromosomal distribution of rice-Arabidopsis WRKY, MADS-box and MYB orthologous gene. (**A**) Distribution of gene loci on *Arabidopsis* chromosomes. (**B**) Distribution of gene loci on rice chromosomes. Different gene loci colours (naming) represents a gene transcription factor family: WRKY; black, MADS-box; purple and MYB; red.

The in silico mapping of WRKY, MADS-box and MYB orthologous gene pairs showed an uneven distribution in *Oryza sativa* (Os) and *Arabidopsis thaliana* (At) chromosomes (Chr). In *Arabidopsis*, the orthologous genes were distributed randomly in AtChr1, AtChr2, AtChr3, AtChr4 and AtChr5. A total of five genes, one from MADS-box, two each from MYB and WRYK TF families were located on AtChr1. On AtChr2 and AtChr4, three WRKY and one MYB genes were located at various distances. All four genes located on AtChr3 are from the MYB family. The AtChr5 showed a random distribution of three MYB and two WRYK genes. In rice, the orthologous genes were present on almost every chromosome except OsChr6, OsChr9 and OsChr10. The OsChr1 contain the highest gene number (GN) at 7, followed by OsChr4 (GN = 3) and OsChr7 (GN = 3), and OsChr8, OsChr11 and OsChr12 with GN = 2 each. The least number of genes were distributed in OsChr2, OsChr3 and OsChr5 (GN = 1) (Fig. 4). Detailed distribution of WRKY, MADS-box and MYB orthologous genes on *Arabidopsis* and rice chromosomes are shown in Table 1. Separated by at least more than five gene loci, no tandem duplications were observed among the genes. The longest protein was encoded by *AtWRKY1* (1789 aa) in *Arabidopsis* and *OsMYB50* (72 aa) in rice. Likewise, the shortest protein was encoded by *AtWRKY43* (109 aa) and *OsWRKY58* (181 aa). More than half of the proteins encoded by *AtMYB* and *OsMYB* genes were acidic with a theoretical isoelectric point value of less than7 whilst two MADS-box proteins (*AtAGL65* and *OsMADS68*) were acidic. A total of 8 *OsWRKY* proteins were acidic in comparison to 2 from *AtWRKY*. The average molecular weight (MW) of these proteins were 48.7 kDa and 45.4 kDa in *Arabidopsis* and rice, respectively. Detailed information on the sequence characteristics is given in Table 1.

**Table 1:** Orthologous WRKY, MADS-box and MYB gene-pairs in *Arabidopsis* and rice.

Gene identifier	Name*	Chr	Location	ORF length (bp)	Protein			Exon number
					Length	PI	Molecular weight (Da)	
AT1G18750	AtAGL65^1^	1	6,466,761–6,469,984	1170	389	6.504	44,877.5	10
AT4G28110	AtMYB41^2^	4	13,968,029–13,969,384	849	282	5.903	31,651.6	3
AT5G56110	AtMYB80^3^	5	22,719,191–22,720,664	963	320	7.322	35,983.4	3
AT3G13540	AtMYB5^4^	3	4,420,173–4,421,701	750	249	8.285	27,793.5	2
AT5G35550	AtMYB123^5^	5	13,726,743–13,727,860	777	258	8.903	29,611.4	3
AT5G12870	AtMYB46^6^	5	4,062,724–4,064,992	843	280	6.037	31,541.3	2
AT1G63910	AtMYB103^7^	1	23,719,783–23,721,774	1113	370	5.681	42,262.6	3
AT3G60460	AtMYB125^8^	3	22,342,429–22,343,491	894	297	6.075	33,649.6	3
AT3G09230	AtMYB1^9^	3	2,833,398–2,835,338	1182	393	5.217	42,811.4	2
AT1G09770	AtMYBCDC5^10^	1	3,161,841–3,165,360	2535	844	6.731	95,766.6	4
AT2G37630	AtMYB91^11^	2	15,781,615–15,783,433	1104	367	9.555	42,243.1	1
AT3G18100	AtMYB4R1^12^	3	6,200,524–6,204,644	2544	847	5.580	96,084.4	7
AT1G29280	AtWRKY65^13^	1	10,236,367–10,237,467	780	259	5.469	29,054.4	2
AT1G68150	AtWRKY9^14^	1	25,543,969–25,545,717	1125	374	7.816	42,743.0	5
AT2G40740	AtWRKY55^15^	2	16,997,177–16,999,277	879	292	8.049	32,488.8	3
AT4G26640	AtWRKY20^16^	4	13,437,071–13,440,835	1458	485	7.102	53,601.5	5
AT4G30935	AtWRKY32^17^	4	15,051,814–15,054,042	1401	466	5.895	51,480.4	5
AT2G37260	AtWRKY44^18^	2	15,644,840–15,647,065	1290	429	9.399	47,141.2	4
AT5G43290	AtWRKY49^19^	5	17,371,838–17,373,201	825	274	7.924	31,580.6	3
AT2G46130	AtWRKY43^20^	2	18,957,226–18,957,911	330	109	9.992	12,951.8	2
AT5G13080	AtWRKY75^21^	5	4,149,740–4,151,150	438	145	9.593	16,801.8	2
AT4G12020	AtWRKY19^22^	4	7,201,656–7,209,648	5397	1798	7.019	199,996.0	15
LOC_Os11g43740	OsMADS68^1^	11	26,414,394–26,418,442	1179	392	6.829	43,366.9	11
LOC_Os07g37210	OsMYB102^2^	7	22,293,735–22,295,309	1107	368	7.092	39,929.0	3
LOC_Os04g39470	OsMYB80^3^	4	23,510,412–23,512,029	1119	372	6.146	39,699.2	3
LOC_Os01g50110	OsMYB13^4^	1	28,796,516–28,797,732	828	275	6.107	29,793.3	2
LOC_Os03g29614	OsMYB46^5^	3	16,879,442–16,883,640	966	321	6.624	34,049	3
LOC_Os12g33070	OsMYB122^6^	12	19,991,426–19,994,401	1230	409	6.824	43,722.4	2
LOC_Os08g05520	OsMYB93^7^	8	2,948,522–2,951,372	1080	359	6.624	39,954.7	3
LOC_Os04g46384	OsMYB58^8^	4	27,503,041–27,504,784	1032	343	7.919	37,110.9	3
LOC_Os01g63160	OsMYB19^9^	1	36,606,535–36,608,135	1242	413	6.697	44,329.6	2
LOC_Os04g28090	OsMYB50^10^	4	16,579,869–16,587,180	2919	972	4.878	109,684	4
LOC_Os12g38400	OsMYB125^11^	12	23,554,928–23,560,551	1029	342	10.28	39,041.6	2
LOC_Os07g04700	OsMYB87^12^	7	2,084,106–2,091,653	2907	968	8.639	106,868.0	13
LOC_Os01g54600	OsWRKY13^13^	1	31,409,004–31,410,978	951	316	4.601	34,294.6	3
LOC_Os02g53100	OsWRKY32^14^	2	32,489,017–32,495,070	1815	604	4.800	62,940.3	6
LOC_Os01g60490	OsWRKY22^15^	1	34,981,468–34,985,447	798	265	7.110	29,807.4	3
LOC_Os07g39480	OsWRKY87^16^	7	23,654,076–23,659,625	1857	618	6.332	66,163.6	6
LOC_Os08g17400	OsWRKY89^17^	8	10,633,195–10,639,603	1653	550	6.707	59,781.9	4
LOC_Os01g62510	OsWRKY119^18^	1	36,188,702–36,191,681	612	203	5.042	21,483.5	2
LOC_Os01g74140	OsWRKY17^19^	1	42,946,753–42,948,750	1233	410	4.685	45,109.9	3
LOC_Os01g53260	OsWRKY23^20^	1	30,604,295–30,608,077	765	254	6.903	27,796.2	2
LOC_Os11g29870	OsWRKY72^21^	11	17,352,085–17,355,820	729	242	9.335	25,857.2	2
LOC_Os05g45230	OsWRKY58^22^	5	26,256,951–26,257,809	546	181	4.631	18,481.3	2

Each gene is described according to chromosome loci, open reading frame (ORF) length, properties of the encoding protein and exon number.

*Similar superscript numbers in the name column represents orthologous gene pairs.

### Gene structure and conserved motif analysis: WRKY, MADS-box and MYB orthologous genes in *Arabidopsis* and rice

**Figure 5:**
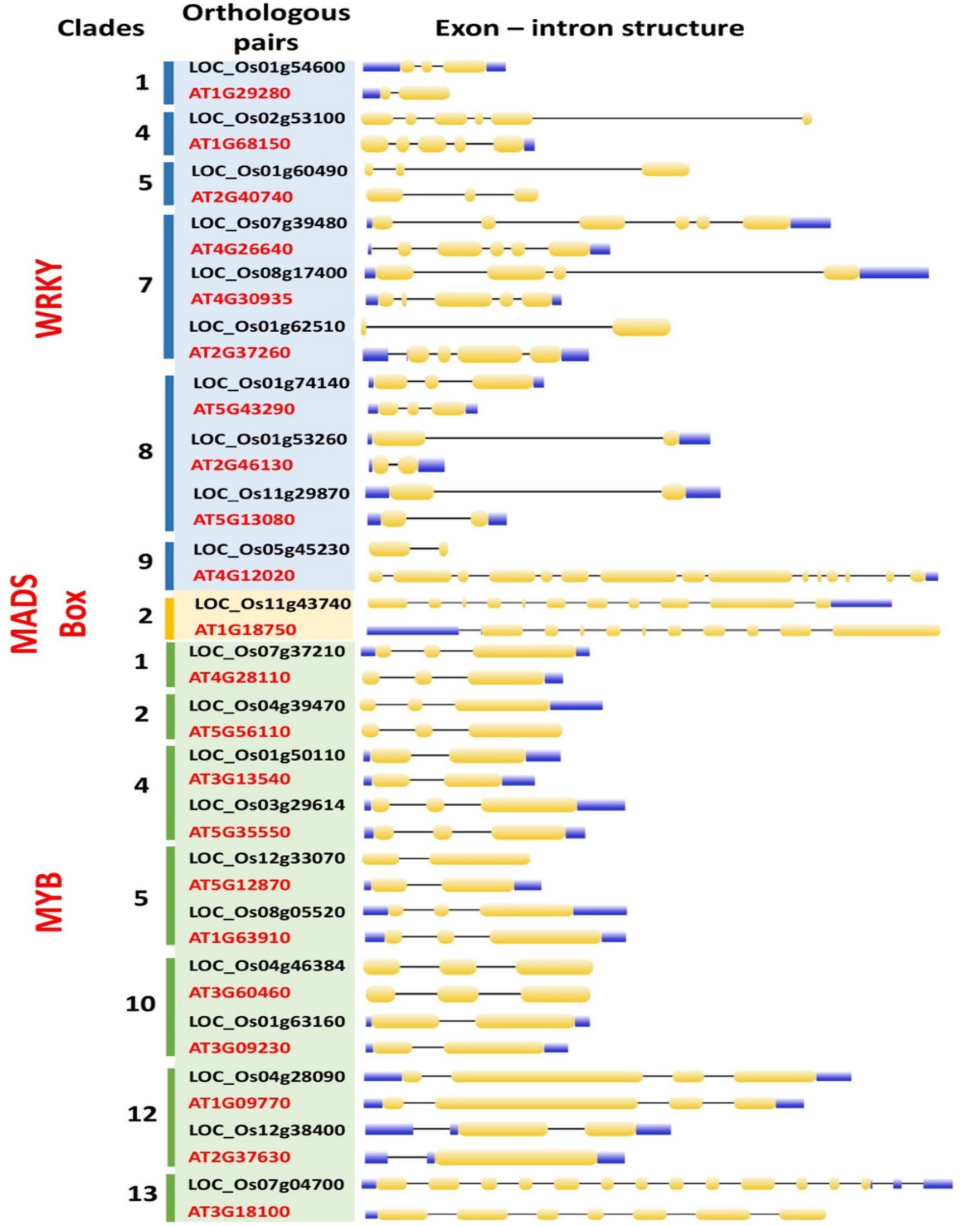
Exon–intron structure of *Arabidopsis* and rice WRKY (blue column), MADS-box (yellow column) and MYB (green column), orthologous gene pairs displayed according to clade numbers in their TF family-phylogenetic tree. The exon–intron structure is described as following: the yellow rectangles and grey lines denote exons and introns, respectively whilst the blue boxes represents the untranslated regions (UTRs).

A total of 173 WRKY, 100 MADS-box and 266 MYB genes were identified with distinctive exon number (EN) and intron number (IN). Among the WRKY genes, EN ranged at 1–15. A total of 95 genes showed EN = 3 and 88 genes showed IN = 3, 22 genes with EN = 2, and 22 genes with EN = 2 and IN = 4. The AT4G12020 gene showed the highest EN and IN with 15 and 14, respectively. Meanwhile, 63 MADS-box genes showed EN = 1, 16 genes with IN = 1, and 13 genes with EN = 2, and eight genes with IN = 2. Among the MYB genes, 156 genes showed EN = 3, 154 genes with IN = 2, 58 genes with EN = 2, and 57 genes with IN = 1 (Supplementary File: Figs. 1, 2, 3). Generally, MADS-box (EN = 1–11) and MYB (EN = 1–13) genes showed a similar range of ENs. Comparatively, the rate of EN and IN difference in the WRKY and MYB TF families was higher than the MADS-box. The exon–intron structure of the ortholog and paralog pairs were further examined. Dissimilarities in the number of exons among the following orthologous gene pairs suggest either a protein gain or loss event in both species: (i) LOC_Os01g54600- AT1G29280, (ii) LOC_Os02g53100- AT1G68150, (iii) LOC_Os11g43740- AT1G18750, and iv) LOC_Os12g38400- AT2G37630. The rice LOC_Os01g54600, LOC_Os02g53100, LOC_Os11g43740 and LOC_Os12g38400 genes were identified to gain one exon whilst their counterpart pairs, AT1G29280, AT1G68150, AT1G18750 and AT2G37630 showed a lost one exon (Fig. 5).

**Figure 6:**
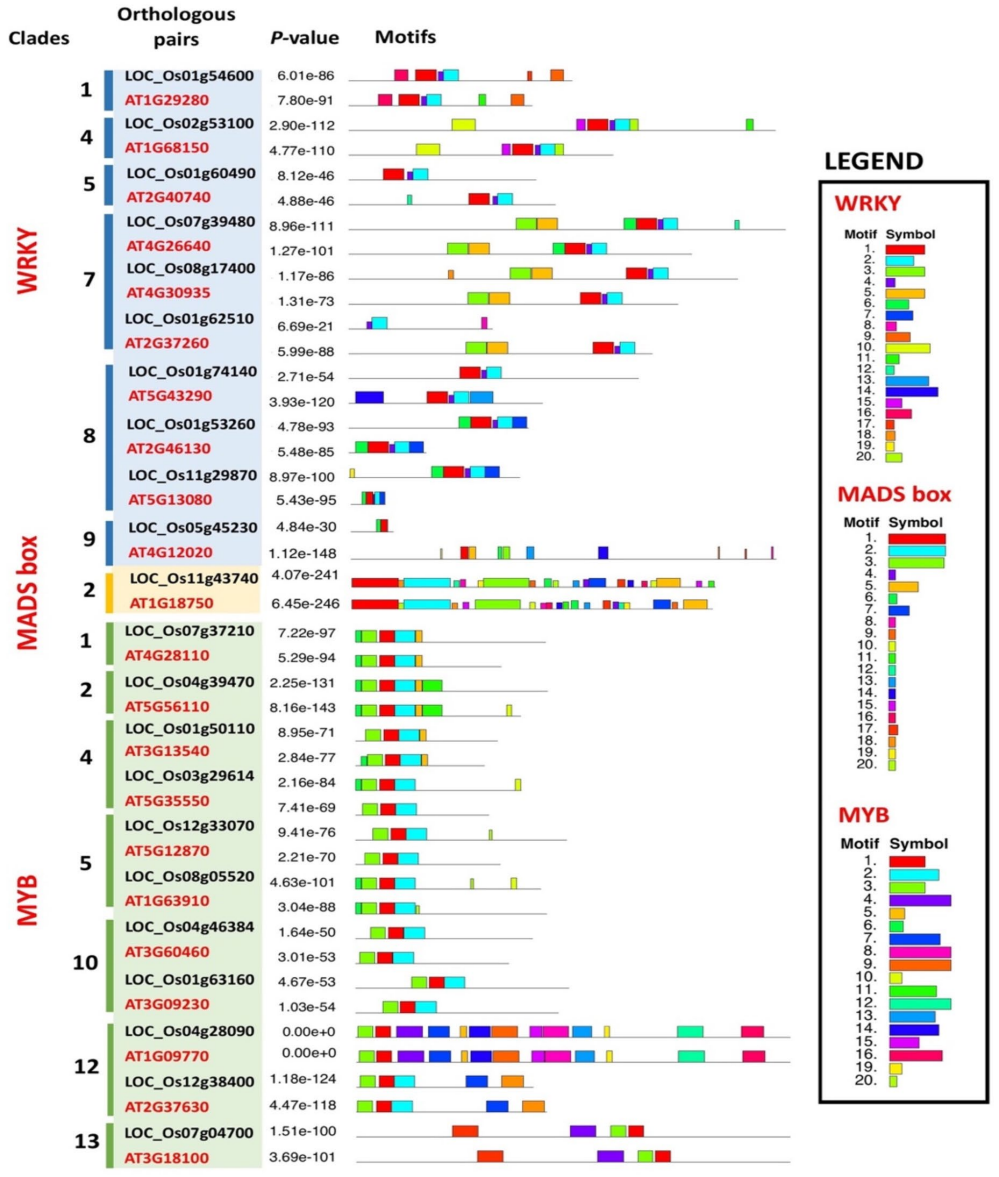
Distribution pattern of conserved motifs in *Arabidopsis* and rice WRKY, MADS-box and MYB orthologous genes, identified by MEME web server. Orthologous gene pairs are presented by transcription factor (TF) families: column blue; WRKY, column yellow; MADS-box and column green; MYB. The *p-*values are significant at 0.05. Motif distribution includes different coloured boxes, each represent a unique numbered motif as indicated in the legend. The width differences among the boxes represents the motif length.

A total of 20 distinct conserved motifs were identified in *Arabidopsis* and rice orthologous genes comprised of 20 WRKY, two MADS-box, and 22 MYB proteins. Almost all orthologous genes, the same type of motifs were present in each gene sequence with different distribution patterns. Evaluation by transcription factor family shows that genes in a common clade shared a closely similar pattern of motif distributions (Fig. 6). The WRKY TF family shows apparent motif similarity with the genes in clade1, 4, 5, 7 and 8 except clade 9. Each clade contains various number of motifs with unique distribution patterns. In the MYB TF family, clade 1–10 were similar with at least 3 identical conserved motifs. Clade 12 showed the highest number of motifs and clade 13 showed the least number. Motif 1 was present within the MYB TF family members whereas motif 2 was found in all clades except clades 12 and 13. The MADS-box TF family represented by a pair of orthologous genes contained 20 different motifs distributed in a similar pattern. Detailed information on motif function annotation of the motifs identified in the WRKY, MYB and MADS-box TF family rice-*Arabidopsis* orthologous genes is presented in Supplementary File 2: Table 1.

### Distribution of *cis*-regulatory elements (CREs) in putative promoter regions of *Arabidopsis* and rice orthologous WRKY, MADS-box and MYB genes

**Figure 7:**
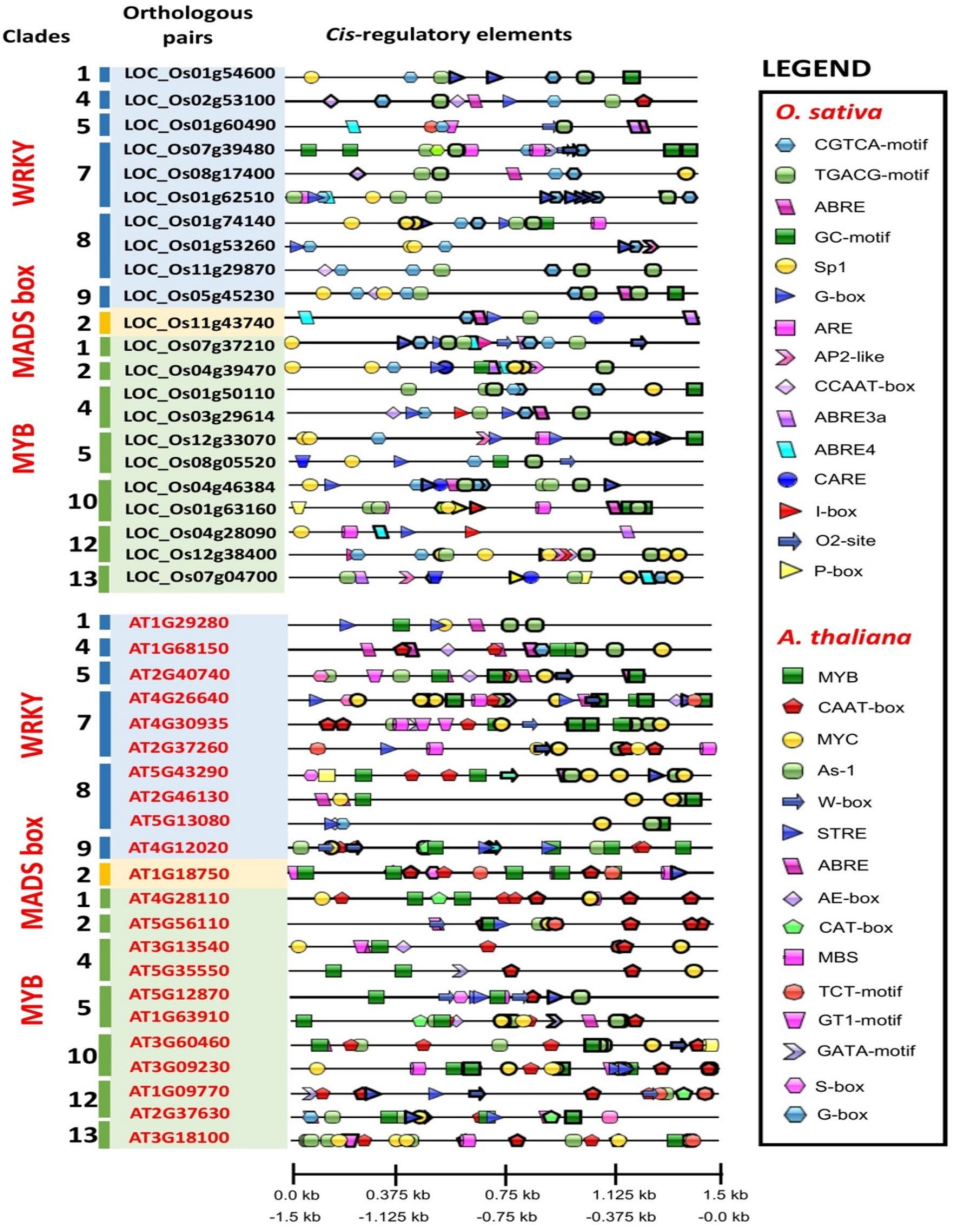
Distribution of the cis-regulatory elements *(*CRE) in the 1.5 kb promoter region of *Arabidopsis* and rice WRKY, MADS box and MYB orthologous genes as identified by PlantCARE and visualized using the IBS software (http://ibs.biocuckoo.org). The CREs are denoted by in different shapes and colours. Each CRE is drawn as following: (i) thick black line for the reverse strand and (ii) thin black line for the forward strand.

The orthologous *Arabidopsis* and rice genes (WRKY, MADS-box and MYB TF family) were screened for cis-regulatory elements (CREs) distribution within the sequence. The CREs were randomly distributed in positive and negative strands of the promoter region of the gene sequence. Comprehensive details of the CREs identified in *Arabidopsis* and rice WRKY, MADS-box and MYB orthologous genes are presented in Supplementary File 4. In rice, the most abundant CREs were encoding for jasmonate-responsive signalling (CGTCA-motif and TGACG-motif), light-responsive (Sp1 and G-box) and plant development (GC-motif) whereas, in *Arabidopsis*, biotic and abiotic stress-responsive elements such as MYB, ABRE, STRE, As-1 and MYC are distributed within the TF family genes. The stress-responsive CRE, ABRE is present in both species, whereas the TGA binding site, such as TGACG-motif and as-1 are unique to rice and *Arabidopsis*, respectively. The CGTCA-motif and TGACG-motif are present in all WRKY, MADS-box and MYB TF family genes except in the *OsMYB50* gene. The MYB binding sites are found in WRKY and MYB genes, with high occurrence in the MYB genes. Other stress-related elements are found in rice genes that include the oxidative stress-responsive element (ARE) and light stress (I-box, Box II and LTR). The elicitor responsive element (W-box), light stress (GT1-motif and GATA-motif) and defense response (G-box) were consistently present in all *Arabidopsis* genes (Fig. 7). The orthologous rice and *Arabidopsis* gene pairs showed common CRE function despite displaying diversity in CRE identities and numbers. The annotation of CREs function involved in the development activities, hormone response and abiotic/biotic stress are compared among the orthologous gene pairs (Table 2).

**Table 2:** Comparison of plant development, hormone and stress-responsive cis-regulatory elements *(*CREs) in the promoter regions of *Arabidopsis* and rice WRKY, MADS-box, and MYB orthologous gene pairs.

Clade	Gene identifier	Name	CRE function		
			Development	Hormone response	Abiotic/biotic stress
2	LOC_Os11g43740	OsMADS68	N//A	CGTCA-motif, TGACG-motif, ABRE, ABRE3a, ABRE4	G-box
	AT1G18750	AtAGL65	N/A	ABRE	S-box, GT1-motif, MBS, MYB, STRE, TCT-motif
1	LOC_Os07g37210	OsMYB102	C-box, O2-site	ABRE3a, ABRE4, CGTCA-motif, TGACG-motif, O2-site	C-box, Sp1
	AT4G28110	AtMYB41	CAT-box	N/A	MBS, MYC, MYB
2	LOC_Os04g39470	OsMYB80	Motif I, AP-2 like	ABRE3a, ABRE4, CGTCA-motif, TGACG-motif	G-box, GC-motif, Sp1
	AT5G56110	AtMYB80	As-1	ABRE, As-1	MYB, MYC, STRE, TCT-motif, W-box
4	LOC_Os01g50110	OsMYB13	N/A	CGTCA-motif, TGACG-motif	GC-motif, Sp1
	AT3G13540	AtMYB5	N/A	ABRE	TCT-motif, MYC, AE-box, GT1-motif, MYB
	LOC_Os03g29614	OsMYB46	N/A	ABRE, CGTCA-motif, TGACG-motif	G-box, I-box, CCAAT-box
	AT5G35550	AtMYB123	N/A	N/A	MYC, GATA-motif, MYB
5	LOC_Os12g33070	OsMYB122	AP-2 like	CGTCA-motif, TGACG-motif	ARE, G-box, GC-motif, I-box, Sp1
	AT5G12870	AtMYB46	As-1	As-1	S-box, MBS, MYB, STRE, W-box
	LOC_Os08g05520	OsMYB93	O2-site	CGTCA-motif, TGACG-motif, O2-site	G-box, GC-motif, Sp1
	AT1G63910	AtMYB103	As-1, CAT-box	ABRE, As-1	AE-box, GATA-motif, MYB, MYC
10	LOC_Os04g46384	OsMYB58	N/A	ABRE, CGTCA-motif, TGACG-motif	G-box, Sp1
	AT3G60460	AtMYB125	As-1	ABRE, As-1	W-box, MYC, MYB, sbp-CMA1c
	LOC_Os01g63160	OsMYB19	GCN4_motif	ABRE, CGTCA-motif, TGACG-motif	ARE, GC-motif, I-box, LTR, P-box, Sp1
	AT3G09230	AtMYB1	As-1	As-1	AE-box, GT1-motif, MBS, MYB, MYC, STRE
12	LOC_Os04g28090	OsMYB50	N/A	ABRE3a, ABRE4	ARE, G-box, P-box, Sp1
	AT1G09770	AtMYBCDC5	As-1, CAT-box	As-1	GATA-motif, STRE, TCT-motif, W-box
	LOC_Os12g38400	OsMYB125	C-box, AP-2 like	CGTCA-motif, TGACG-motif	C-box, CCAAT-box, G-box, Sp1
	AT2G37630	AtMYB91	As-1, CAT-box	ABRE, JERE	AE-box, MYB, MYC, STRE
13	LOC_Os07g04700	OsMYB87	AP-2 like	ABRE3a, ABRE4, CGTCA-motif, TGACG-motif	LTR, P-box, Sp1
	AT3G18100	AtMYB4R1	As-1	As-1	GT1-motif, MBS, MYB, MYC, TCT-motif
1	LOC_Os01g54600	OsWRKY13	N/A	CGTCA-motif, TGACG-motif	GC-motif, G-box
	AT1G29280	AtWRKY65	As-1	ABRE, As-1	MYB, MYC, STRE
4	LOC_Os02g53100	OsWRKY32	N/A	ABRE, CGTCA-motif, TGACG-motif	G-box, CCAAT-box, Sp-1
	AT1G68150	AtWRKY9	As-1	ABRE, As-1	AE-box, G-box, MYB, MYC
5	LOC_Os01g60490	OsWRKY22	O2-site	ABRE, ABRE3a, ABRE4, CGTCA-motif, TGACG-motif, O2-site	Box II
	AT2G40740	AtWRKY55	As-1, CAT-box	ABRE, As-1	AE-box, S-box, GT1-motif, MYB, MYC, W-box
7	LOC_Os07g39480	OsWRKY87	GCN4_motif, O2-site	CGTCA-motif, TGACG-motif, O2-site	ARE, GC-motif
	AT4G26640	AtWRKY20	As-1	ABRE, As-1	AE-box, S-box, G-box, GATA-motif, MBS, MYB, MYC, STRE, TCT-motif, W-box
	LOC_Os08g17400	OsWRKY89	O2-site	ABRE, CGTCA-motif, TGACG-motif, O2-site	CCAAT-box, Sp1
	AT4G30935	AtWRKY32	As-1	As-1	GATA-motif, GT1-motif, MBS, MYB, MYC, W-box
	LOC_Os01g62510	OsWRKY119	N/A	ABRE3a, ABRE4, CGTCA-motif, TGACG-motif	ARE, G-box, GC-motif, Sp1
	AT2G37260	AtWRKY44	As-1	As-1	MBS, MYC, STRE, TCT-motif, W-box
8	LOC_Os01g74140	OsWRKY17	N/A	CGTCA-motif, TGACG-motif	ARE, G-box, Sp1, GC-motif
	AT5G43290	AtWRKY49	As-1	ABRE, As-1	S-box, DRE core, Gap-box, MYB, MYC, STRE
	LOC_Os01g53260	OsWRKY23	AP-2 like	CGTCA-motif, TGACG-motif	G-box, Sp1
	AT2G46130	AtWRKY43	As-1	ABRE, As-1	MYB, MYC
	LOC_Os11g29870	OsWRKY72	N/A	CGTCA-motif, TGACG-motif	CCAAT-box
	AT5G13080	AtWRKY75	As-1	As-1	MYC, MYB, AE-box, G-box, STRE
9	LOC_Os05g45230	OsWRKY58	N/A	ABRE, CGTCA-motif, TGACG-motif	CCAAT-box, GC-motif, Sp1
	AT4G12020	AtWRKY19	As-1, CAT-box	As-1	DRE core, MYB, MYC, STRE, W-box

### In silico analysis of co-expression and functional similarity between *Arabidopsis* and rice orthologous gene pairs

**Figure 8:**
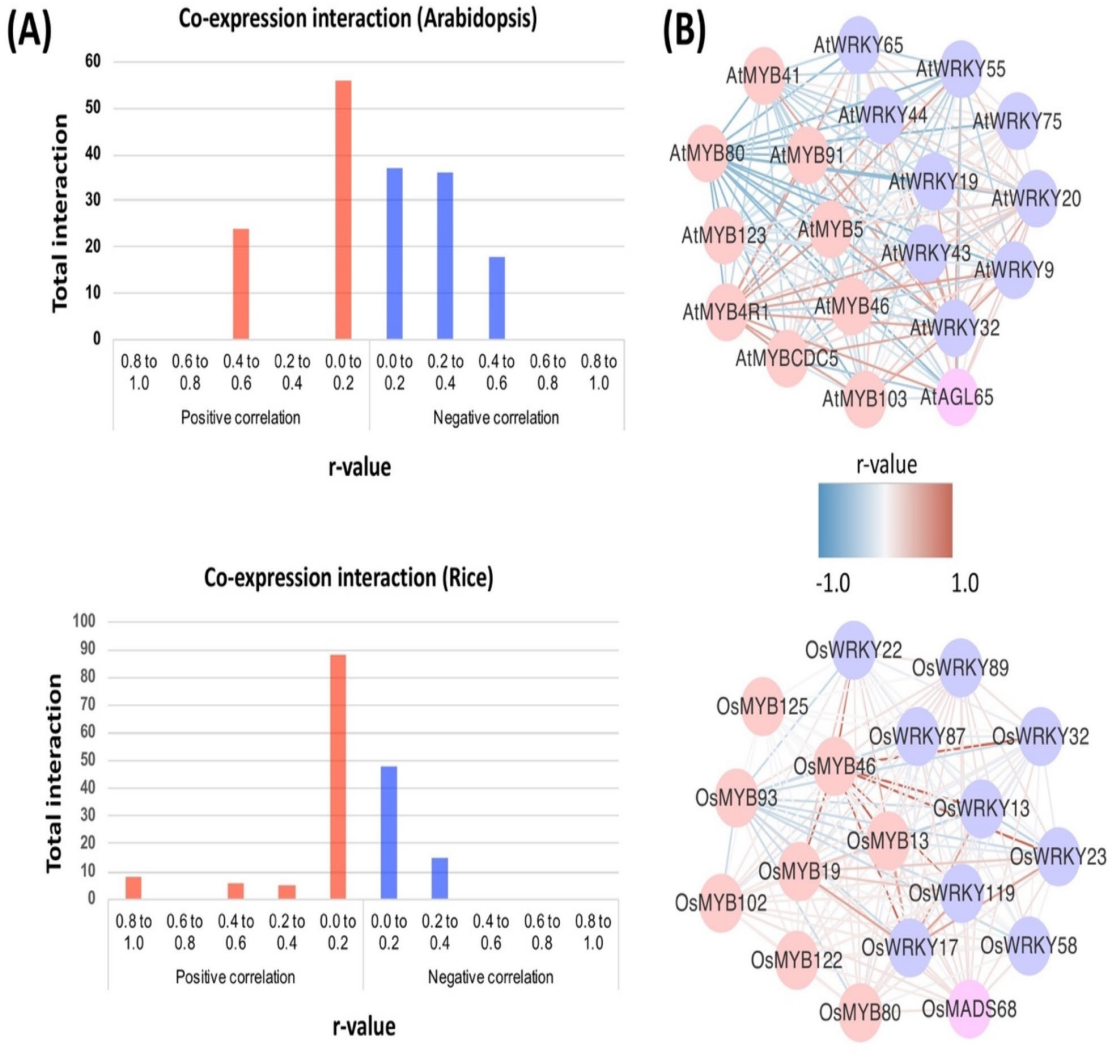
Gene co-expression network of *Arabidopsis* and rice WRKY, MADS-box and MYB orthologous genes. (**A**) Frequencies of co-expression interactions identified by PLANEX. Increasing r-values show stronger positive correlation and vice versa. (**B**) Co-expression network comprised of nodes, represent genes, different node colour s indicate unique transcription factor family (red node = MYB, blue node = WRKY and purple node = MADS-box) and edges indicate positive (red lines) and negative (blue lines) correlations.

Co-expression analysis was conducted on the 19 *Arabidopsis* and 18 rice orthologous genes identified in the previous analysis where the expression datasets were retrieved from PLANEX (planex.plantbioinformatics.org). The correlation values (r) among the WRKY, MADS-box and MYB genes in *Arabidopsis* and rice were ranked as follows: (i) poor; r < 0.20, (ii) fairly moderate; r = 0.2–0.4, (iii) fairly strong; r > 0.4–0.6 and (iv) strong; r > 0.6–0.8. The average positive correlation within the *Arabidopsis* and rice network were 0.212 and 0.160, respectively. The negative correlation of the *Arabidopsis* network (r = − 0.248) was much stronger than the rice network(r = − 0.084). In *Arabidopsis*, *AtMYB4R1* showed the strongest correlation (r = 0.465, fairly strong) with MADS-box (*AtAGL65*), MYB (*AtMYB103*, *AtMYB91*, *AtMYB5* and *AtMYBCDC5*) and WRKY (*AtWRKY65*, *AtWRKY9*, *AtWRKY44*, *AtWRKY55* and *AtWRKY43*) transcription factor (TF) genes. For rice TFs, *OsMYB46* showed the strongest correlation with *OsMYB13*, *OsMYB19*, *OsWRKY13*, *OsWRKY17*, *OsWRKY22*, *OsWRKY23*, *OsWRKY32* and *OsWRKY119* shown at r = 0.827 (Fig. 8).

The occurrence of possible functional similarity between *Arabidopsis* and rice orthologous genes were compared on their co-expression networks using the Kappa statistics retrieved from PLANEX (Table 3). Kappa (*k*) score = 1 denotes a perfect functional similarity between networks^35,38^. A *k*-score > 0 is assumed significantly similar, whilst *k-*score = 0 denotes no significant similarity^35,38^. Eleven *Arabidopsis*-rice orthologous genes were accounted for 69% of the total genes (*k-*score = 0.2 – 0.4) that showed fair functional similarity, followed by three genes (19%) and two genes (13%) of poor (*k-*score =  > 0.0 to 0.2) and moderate (*k-*score = 0.4 to 0.6) functional similarity, respectively. The *OsWRKY32*- *AtWRKY9* and *OsMADS68*-*AtAGL65* orthologous pairs were highly significant with a *k-*score of 0.44 and 0.50, respectively.

**Table 3:** Functional similarity between the *Arabidopsis* and rice WRKY, MADS-box and MYB orthologous gene-pairs.

Rice			*Arabidopsis*			Kappa statistics
Gene ID	Name	Probe ID	Gene ID	Name	Probe ID	
LOC_Os11g43740	OsMADS68	OsAffx.19355.1.S1_at	AT1G18750	AtAGL65	261423_at	0.500029866483831
LOC_Os07g37210	OsMYB102	Os.3390.1.S1_at	AT4G28110	AtMYB41	253851_at	0.236403501449661
LOC_Os04g39470	OsMYB80	OsAffx.14205.1.S1_at	AT5G56110	AtMYB80	248051_at	0.162086262661477
LOC_Os01g50110	OsMYB13	Os.55528.1.S1_at	AT3G13540	AtMYB5	256985_at	0.342306085866936
LOC_Os03g29614	OsMYB46	Os.56985.1.S1_a_at	AT5G35550	AtMYB123	249704_at	0.355608075400779
LOC_Os12g33070	OsMYB122	OsAffx.19945.2.S1_at	AT5G12870	AtMYB46	250322_at	0.122141588277817
LOC_Os08g05520	OsMYB93	Os.49830.1.S1_at	AT1G63910	AtMYB103	260326_at	0.316581470795973
LOC_Os12g38400	OsMYB125	Os.12994.1.S1_at	AT2G37630	AtMYB91	267157_at	0.348381644725455
LOC_Os01g54600	OsWRKY13	Os.2160.2.S1_x_at	AT1G29280	AtWRKY65	260882_at	0.293798964280973
LOC_Os02g53100	OsWRKY32	OsAffx.12620.1.S1_at	AT1G68150	AtWRKY9	260432_at	0.439323046291688
LOC_Os01g60490	OsWRKY22	OsAffx.23871.1.S1_at	AT2G40740	AtWRKY55	266052_at	0.309001957815947
LOC_Os07g39480	OsWRKY87	Os.18862.1.S1_at	AT4G26640	AtWRKY20	253983_at	0.263336526419911
LOC_Os08g17400	OsWRKY89	Os.27818.1.S1_at	AT4G30935	AtWRKY32	253603_at	0.206776541657964
LOC_Os01g62510	OsWRKY119	OsAffx.9554.1.S1_at	AT2G37260	AtWRKY44	265954_at	0.185878341728013
LOC_Os01g53260	OsWRKY23	Os.30386.1.S1_at	AT2G46130	AtWRKY43	266597_at	0.372919466881054
LOC_Os05g45230	OsWRKY58	OsAffx.27315.1.S1_at	AT4G12020	AtWRKY19	254852_at	0.208021020730278

The co-expression datasets are retrieved and analyzed using Kappa statistics from PLANEX.

## Discussion

Over the years, natural and human activities have caused significant changes to the global environment. Climate change, decrease in arable land, increase in CO_2_ concentration, declining water availability, drought and high salinity had set major challenges to agricultural systems, worldwide. The quest for yield and productivity is becoming increasingly challenging with a continuum decline in plant stress resistance. Plants are complex multicellular organisms with highly flexible adaptivity to adverse conditions such as the exposure to abiotic and biotic factors that trigger various responses governed by complex regulatory mechanism i.e. the transcriptional regulation^39^ and through gene expression, they respond to these changes by either activating or repressing the expression of the downstream genes^40,41^.

Transcription factors (TFs) are deployed as the master key regulators in plant growth and development, and defense-related responses. The WRKY, MADS-box and MYB are major TF families that regulate various aspects of plant development through specificity and/or crosstalk regulation between different TFs; growth and developmental processes^42^, and biotic and abiotic stress responses^35,43,44^. Cis-acting regulatory elements (CREs) at the binding site or near to the structural genes interact with TFs to control the expression of the corresponding genes. The promoters present at the upstream of a gene encoded region contain numerous CREs which are unique to various proteins involved in the transcription initiation and regulation^40,45^. The CREs have been reported to display diverse functions associated with biotic and abiotic components: pathogen and wound responsive, light and phytohormone responsive. Studies on *cis-*regulatory elements (CREs) are important to further understand the plant defense responses to abiotic and biotic stresses ^38^.

In this study, the *Arabidopsis* and rice WRKY, MADS-box and MYB TF genes showed a similar TF-family abundance level. Although the rice genome size is larger than *Arabidopsis’s*, the number of TF genes in both species were similar. Phylogenetic trees built on a collated rice and *Arabidopsis* WRKY, MADS-box and MYB TF family members were each divided into 10, 7 and 14 clades, respectively. The findings suggest that MYB TF family is the most diverse family, followed by WRKY and the MADS-box, being the least diverse TF family. Generally, both WRKY and MYB TF members were much closely related to one other in comparison to MADS-box members. In the WRKY- and MYB- specific phylogenetic tree, both the *Arabidopsis* and rice genes were present in virtually all clades. In contrast, MADS-box specific-phylogenetic tree, very few clades showed a representation of rice and Arabidopsis; clades were dominated by a single species, either the *Arabidopsis* or rice (Fig. 2). Ortholog genes are similar genes with the same gene function that may have arisen from speciation events. A relatively higher number of orthologous gene pairs observed in the *Arabidopsis*-rice WRKY and MYB TF families may explain the existence of ancestral relationships between *Arabidopsis* and rice before divergence during evolution (Figs. 1 and 3). Chromosomal distribution of orthologous WRKY and MYB genes in rice and *Arabidopsis* showed no apparent pattern. However, it is noteworthy to mention that most of the orthologous genes were distributed within the single arms of the chromosomes (Fig. 4).

Gene structure analysis imparts understanding into evolutionary processes such as duplication events^46^. In this study, the three different TF orthologous gene families from *Arabidopsis* and rice displayed various exon and intron numbers, implying possible roles in diversification events of the two Angiosperms. For instance, the rice *OsWRKY13* gene consists of three exons, whilst its counterpart orthologous pair, the *Arabidopsis AtWRKY65* contains two exons only. These results suggest that some of the TF family genes may have undergone loss of introns during the evolutionary processes and cause subsequent functional differences in rice and *Arabidopsis*. Most of the *Arabidopsis*-rice orthologous gene pairs under the WRKY and MYB TF family consist of similar exon numbers, and thus, implies similar gene function acquirement during stable evolution^47^. The number of proteins with motifs identified in the WRKY TF family was comparable to the MYB TF family; 20–22 proteins. The MADS-box TF family contained only two protein sequences with motifs (Fig. 6). The disparity between the WRKY and MYB TF families over the MADS-box TF family could be implicated in the functional differences between these TF families. The MADS-box are highly involved in plant growth and development in comparison to WRKY and MYB TF families which are actively responsive to biotic and abiotic responses. Similar types of motifs were identified in all three TF families, however, the motif and CRE distribution displayed a similar trend by the TF family suggesting the functional niche unique to each TF family.

Motif distributions are conserved between the orthologous gene pairs that share a common clade. Each specific motif present in the orthologous genes corresponds to a specific protein function. For example, WRKY genes with a DNA-binding domain were mainly enriched within motif 1–3 and 5. MYB genes enriched with motif 1–4 correspond to Myb-like DNA-binding domain and MADS-box genes with an abundant number of motif 1, motif 3 and motif 5 correspond to DNA-binding and dimerisation domain, K-box region and connexin4, respectively. In general, WRKY and MYB orthologous genes show motif abundance and diversity to a major extent. It is also noteworthy to observe the impact of motif loss in the orthologous gene pairs. As such, the rice *OsWRKY58* gene lacks motifs 5, 6, 9, 10, 13, 14, 15 and 18 in comparison to its orthologous pair, which is the *Arabidopsis AtWRKY19* gene. These differences may imply the occurrence of the *OsWRKY58* gene functional divergence with the *AtWRKY19* gene*.*

The CRE analysis of *Arabidopsis* and rice WRKY, MADS-box and MYB genes showed functional involvement in stress-related, phytohormone-related and plant development-related activities. All *Arabidopsis* and rice genes contain a combination of different CREs except for the following orthologous pairs which contain a phytohormone-related ABRE motif: *OsMADS68-AtAGL65* (clade 2, MADS-box TF), *OsMYB58*-*AtMYB125* (clade 10, MYB TF) and *OsWRKY22-AtWRKY55* (clade 5, WRKYT F). The *OsWRKY32-AtWRKY9* orthologous pair share both ABRE and G-box element motifs. Previous studies showed the role of G-box as a stress-responsive element against pathogen^48^, in phytohormone like abscisic acid (ABA) and jasmonic acid (JA) signalling regulator, and favours reactive oxygen species (ROS) burst under environmental stress^47,49^. Additionally, ABA responsive element (ABRE) also acts as a positive regulator of ABA signalling under saline and drought conditions^47,50^. Phytohormone-related elements (CGTCA-motif and TGACG-motif) abundantly present in rice genes suggest its crucial function in JA-responsiveness. The TGACG-motif and As-1 elements are both known as TGA elements. Interestingly, TGACG-motif was predominantly found in rice genes and As-1 element in *Arabidopsis* genes, mainly. Our findings showed an apparent divergence of stress-related elements in rice and *Arabidopsis*. The CREs that are unique to rice genes are Sp1, ARE and GC-motif. On the other hand, MYB, MYC, STRE and W-box motifs are unique to the *Arabidopsis* gene. ARE (Anaerobic responsive elements) consisting of GC and GT motifs act as an oxidative responsive element. Previous studies showed that the rice genome contains higher GC motifs than in *Arabidopsis*^47,51^.

An ongoing duplication event within plant species may had led to the divergence of the WRKY, MADS-box and MYB TF families. Apparent gain and loss in gene structures were evident within each TF family. Co-expression network analysis revealed a moderately fair (r = 0.2–0.4) interaction in *Arabidopsis* and poor interaction(r =  > 0–0.2) in rice. *OsMYB46* gene in rice encodes the transcriptional regulation of secondary wall biosynthesis. Rice co-expression network analysis has shown a strong association of the *OsMYB46* gene with lignin biosynthetic transcription factors (*OsMYB13* and *OsMYB19)*^52^, and rice resistance to blast and bacterial blight encoding *OsWRKY22*^53^, *OsWRKY13*^54^ and *OsWRKY23*^55^ genes. These findings suggest that both MYB and WRK TF family genes are switched on to orchestrate SA- and JA- mediated signalling pathways during the pathogen attack.

The functional similarities between WRKY, MADS-box and MYB genes within *Arabidopsis* and rice was measured and compared against each other via the co-expression network analysis. Two independent *Arabidopsis* and rice co-expression networks were about similar size as indicated by the total number of nodes (number of genes); 19 in Arabidopsis and 18 in the rice co-expression network. In each co-expression network, all three different WRKY, MADS-box and MYB genes showed positive and negative correlations to a considerable extent. Interestingly, the hub gene denotes as the gene with the most number of interactions belongs to the MYB TF family in both Arabidopsis and rice co-expression networks.

The functional similarities of *Arabidopsis* and rice orthologous gene- pairs were detected at significant k-scores^38^. Previously studies using co-expression networks analysis have functionally characterized several genes, i.e. the *Arabidopsis AtAGL65* gene that regulates pollen tube growth and maturation^56^, and *OsMADS68* that regulates the downstream *OsCPK21* gene during anther development in rice^57^. The *OsMYB80-AtMYB80*, rice-*Arabidopsis* orthologous gene pair is functionally conserved as the positive regulators of pollen development^58,59^. Meanwhile, the *Arabidopsis AtWRKY9* gene was shown to be induced in response to pathogen-associated molecular patterns (PAMP)^52^, and the rice *OsWRKY32* gene has been activated during rice blast pathogen, *Magnoporthae oryzae* pathogenesis^60^. Based on the expression profiles, *Arabidopsis AtWRKY43* gene showed close association with the pathogen defense transcription factor, the rice *OsWRKY23* gene^55,61^. The discovery of stress-related genes and their association with the *Arabidopsis* and rice WRKY, MADS-box and MYB orthologous genes offers a basis for future biotechnology and breeding studies aimed to enhance plant stress responses.

Feeding more than half the world population, rice is a premier staple food worldwide, especially among the majority of Asians. Rice yield improvement has been a key breeding objective as farming and subsequent productivity are affected by numerous factors such as soil fertility, abiotic stressors (salinity, drought, heat and cold) and susceptibility to a wide range of diseases. The present-day rice breeding strategies have evolved tremendously. From conventional breeding to breeding by design, the identification of candidate desirable genes is a core component to kickstart any breeding programmes. Improvement of complex traits controlled by multiple genes with each displaying a relatively small effect had led to trait-based selections that are unfavourably related^62^. As a result, the current pace of rice breeding does not meet the breeding objectives designed for the development of climate-resilient, fit and adaptive, and resource-use efficient cultivars.

Gene similarities are key aspect of gene function. Gene data sets which includes the gene expression and gene co-expression networks elucidate associated functions between genes across and within the plant kingdoms. The overall functional similarity between two genes requires multi-aspect considerations. Although both rice and Arabidopsis are two important model plant organisms subjected to different research pace, the latter is much more thoroughly investigated and functionally described in comparison to rice. In addition, most gene function association studies performed are projected on Arabidopsis to better understand the any given plant organism of interest. In this study, the Arabidopsis and rice TF families are comparatively evaluated to gain multi-dimensional information on the WRKY, MADS-box and MYB gene pattern of distribution, structure and function.

In the ‘breeding by design’ technique such as the target chromosome-segment substitution^63^, mapping of loci governing agronomically desirable traits serves as the pre-requisite step. Under this technique, information on the desirable gene loci along their interrelated functional roles is crucial to accomplish a successful breeding programme. Ultimately, using transcription factor genes, the present findings offer a knowledge base to facilitate efficient selection of desirable genes as TF genes among the different families (WRKY, MADS-box and MYB) displaying inter-relations with each other. In parallel, current findings enables manipulation of biologically important multi-functional TF genes governing rice stress responses and developmental processes. Rice improvement guards global food security, and thus, the production of resilient planting materials could be facilitated and accelerated in breeding programmes catered for rapid development of rice varieties.

## Conclusions

Plant growth and development, and environmental responses are key targets for manipulation in biotechnology and breeding programmes. This study investigated 172 WRKY, 100 MADS-box and 266 MYB TF genes in *Arabidopsis* and rice. Twenty-two *Arabidopsis*-rice orthologous gene pairs were identified from the WRKY, MADS-box and MYB TF family, and their exon–intron distribution along the motif compositions are mostly similar and conserved. The majority of the WRKY, MADS-box and MYB genes in *Arabidopsis* and rice showed specific interaction with abiotic/biotic and phytohormone responsiveness elements. Further, the co-expression interaction among the WRKY, MADS-box and MYB genes between *Arabidopsis* and rice illustrated a similar trend based on the average correlation measurement. The functional similarity of co-expression data comprised of orthologous genes indicates their important roles in pollen development, hormone-mediated and defense response to the pathogen. The orthologous genes identified in this study informs the selection of genes governing the conserved regulatory module of defense and development in rice and *Arabidopsis.*

## Supplementary Information

Supplementary Information 1.

Supplementary Information 2.

Supplementary Information 3.

Supplementary Information 4.

## References

[1] Hao LY (2020). Genome-wide identification and comparative analysis of drought related genes in roots of two maize inbred lines with contrasting drought tolerance by RNA sequencing. J. Integr. Agric..

[2] Mishra P (2021). In silico mining of WRKY TFs through *Solanum melongena* L. and *Solanum incanum* L. transcriptomes and identification of SiWRKY53 as a source of resistance to bacterial wilt. Plant Gene.

[3] Balaguer MADL (2017). Predicting gene regulatory networks by combining spatial and temporal gene expression data in *Arabidopsis* root stem cells. Proc. Natl. Acad. Sci. U. S. A..

[4] Joshi R (2016). Transcription factors and plants response to drought stress: Current understanding and future directions. Front Plant Sci..

[5] Srivastava R (2018). Comparative genome-wide analysis of WRKY transcription factors in two Asian legume crops: Adzuki bean and Mung bean. Sci. Rep..

[6] Wu KL, Guo ZJ, Wang HH, Li J (2005). The WRKY family of transcription factors in rice and *Arabidopsis* and their origins. DNA Res..

[7] Rushton PJ, Somssich IE, Ringler P, Shen QJ (2010). WRKY transcription factors. Trends Plant Sci..

[8] Yanhui C (2006). The MYB transcription factor superfamily of Arabidopsis: Expression analysis and phylogenetic comparison with the rice MYB family. Plant Mol. Biol..

[9] Li C, Ng CKY, Fan LM (2015). MYB transcription factors, active players in abiotic stress signaling. Environ. Exp. Bot..

[10] Ogatallz K (1996). The cavity in the hydrophobic core of Myb DNA-binding domain is reserved for DNA recognition and trans-activation. Nat. Struct. Biol..

[11] Jia L, Clegg MT, Jiang T (2004). Evolutionary dynamics of the DNA-binding domains in putative R2R3-MYB genes identified from rice subspecies indica and japonica genomes. Plant Physiol..

[12] Leng B (2021). Heterologous expression of the *Limonium bicolor* MYB transcription factor LbTRY in *Arabidopsis thaliana* increases salt sensitivity by modifying root hair development and osmotic homeostasis. Plant Sci..

[13] Initiative TAG (2000). Analysis of the genome sequence of *Arabidopsis thaliana*. Nature.

[14] Wixon J (2001). Arabidopsis thaliana. Int. J. Genomics.

[15] Jin J (2016). PlantTFDB 4.0: Toward a central hub for transcription factors and regulatory interactions in plants. Nucl. Acids Res..

[16] Tiwari P (2020). Auxin-salicylic acid cross-talk ameliorates OsMYB-R1 mediated defense towards heavy metal, drought and fungal stress. J. Hazard Mater..

[17] Park DY (2018). The MYB- related transcription factor RADIALIS-LIKE3 (OsRL3) functions in ABA-induced leaf senenscence and salt sensitivity in rice. Environ. Exp. Bot..

[18] Wang P (2018). OsWRKY28 regulates phosphate and arsenate accumulation, root system, architecture and fertility in rice. Front. Plant Sci..

[19] Dai X, Wang Y, Zhang WH (2016). OsWRKY74, a WRKY transcription factor, modulates tolerance to phosphate starvation in rice. J. Exp. Bot..

[20] Chen H (2018). OsMADS27 regulates the root development in a NO3-dependent manner and modulates the salt tolerance in rice (*Oryza sativa* L.). Plant Sci..

[21] Yuan F, Xu Y, Leng B, Wang B (2019). beneficial effects of salt on halophyte growth: morphology, cells and genes. Open Life Sci..

[22] Smaczniak C, Immink RG, Angenent GC, Kaufmann K (2012). Developmental and evolutionary diversity of plant MADS-domain factors: insights from recent studies. Development.

[23] Goodstein DM (2012). Phytozome: A comparative platform for green plant genomics. Nucl. Acids Res..

[24] Thompson JD, Gibson TJ, Higgins DG (2002). Multiple sequence alignment using ClustalW and ClustalX. Curr. Protoc. Bioinform..

[25] Saitou N, Nei M (1987). The neighbor-joining method: A new method for reconstructing phylogenetic trees. Mol. Biol. Evol..

[26] Gascuel O, Steel M (2006). Neighbor-joining revealed. Mol. Biol. Evol..

[27] Rambaut, A. FigTree v1.4.3:*Molecular Evolution, Phylogenetics and Epidemiolog*; 2007 Updated (2016).

[28] Rhee SY (2003). The Arabidopsis Information Resource (TAIR): A model organism database providing a centralized, curated gateway to *Arabidopsis* biology, research materials and community. Nucl. Acids Res..

[29] Kurata N, Yamazaki Y (2006). Oryzabase. An integrated biological and genome information database for rice. Plant Physiol..

[30] Wang H, Zhao S, Gao Y, Yang J (2017). Characterization of dof transcription factors and their responses to osmotic stress in poplar (*Populus trichocarpa*)”. PLoS ONE.

[31] Hu B, Jin J, Guo A, Zhang H, Luo J (2014). GSDS 2.0: An upgraded gene feature visualization server. Bioinformatics.

[32] Bailey TL, Johnson J, Grant CE, Noble WS (2015). The MEME suite. Nucl. Acids Res..

[33] Finn RD (2014). Pfam: the protein families database. Nucl. Acids Res..

[34] Lescot M (2002). PlantCARE, a database of plant cis-acting regulatory elements and a portal to tools for in silico analysis of promoter sequences. Nucl. Acids Res..

[35] Chen C (2016). Heat stress yields a unique MADS box transcription factor in determining seed size and thermal sensitivity. Plant Physiol..

[36] Yim WC, Yu Y, Song K, Jang CS, Lee BM (2013). PLANEX: The plant co-expression database. BMC Plant Biol..

[37] Shannon P (2003). Cytoscape: A software environment for integrated models of biomolecular interaction networks. Genome Res..

[38] Kaur A, Pati PK, Pati AM, Nagpal AK (2017). *In-silico* analysis of cis-acting regulatory elements of pathogenesis-related proteins of *Arabidopsis thaliana* and *Oryza sativa*. PLoS ONE.

[39] Maag D, Erb M, Köllner D, Gershenzon J (2014). Defensive weapons and defense signals in plants: Some metabolites serve both roles. BioEssays.

[40] Ho CL, Geisler M (2019). Genome-wide computational identification of biologically significant Cis-regulatory elements and associated transcription factors from rice. Plants (Basel).

[41] Mulat MW, Sinha VB (2020). Identification and characterization of Dof in Tef[Eragrostis tef (Zucc.) Troetter]. using in silico approaches. Gene Rep..

[42] Ramamoorthy R, Jiang S, Kumar N, Venkatesh PN, Ramachandran S (2018). A comprehensive transcriptional profiling of the WRKY gene family in rice under various abiotic and phytohormone treatments. Plant Cell Physiol..

[43] Wu TY (2020). Crosstalk between heterotrimeric G protein-coupled signaling pathways and WRKY transcription factors modulating plant responses to suboptimal micronutrient conditions. J. Exp. Bot..

[44] Jiang J (2017). WRKY transcription factors in plant responses to stresses. J. Integr. Plant Biol..

[45] Mulat MW, Sinha VB (2021). Distribution and abundance of CREs in the promoters depicts crosstalk by WRKYs in Tef[Eragrostic tef (Zucc) Troetter]. Gene Rep..

[46] Yang A, Dai X, Zhang WH (2012). A R2R3-type MYB gene, OsMYB2, is involved in salt, cold, and dehydration tolerance in rice. J. Exp. Bot..

[47] Dai X (2007). Overexpression of an R1R2R3 MYB gene, OsMYB3R-2, increases tolerance to freezing, drought, and salt stress in transgenic Arabidopsis. Plant Physiol..

[48] Kong W, Ding L, Cheng J, Wang B (2018). Identification and expression analysis of genes with pathogen-inducible cis-regulatory elements in the promoter regions in *Oryza sativa*. Rice.

[49] Ramegowda V (2017). GBF3 transcription factor imparts drought tolerance in *Arabidopsis thaliana*. Sci. Rep..

[50] Lu Y (2019). Genome-wide identification and expression analysis of glycine-rich RNA-binding protein family in sweet potato wild relative *Ipomoea trifida*. Gene.

[51] Ferguson AA, Jiang N (2011). Pack-MULEs: recycling and reshaping genes through GC-biased acquisition. Mob. Genet. Elements.

[52] Zhong R (2011). Transcriptional activation of secondary wall biosynthesis by rice and maize NAC and MYB transcription factors. Plant Cell Physiol..

[53] Abbruscato P (2012). OsWRKY22, a monocot wrky gene, plays a role in the resistance response to blast. Mol. Plant Pathol..

[54] Qiu D (2007). OsWRKY13 mediates rice disease resistance by regulating defense-related genes in salicylate- and jasmonate-dependent signaling. Mol. Plant-Microbe Interact..

[55] Jing S, Zhou X, Song Y, Yu D (2009). Heterologous expression of OsWRKY23 gene enhances pathogen defense and dark-induced leaf senescence in Arabidopsis. Plant Growth Regul..

[56] Adamczyk BJ, Fernandez DE (2009). MIKC* MADS domain heterodimers are required for pollen maturation and tube growth in Arabidopsis. Plant Physiol..

[57] Wen K (2019). OsCPK21 is required for pollen late-stage development in rice. J. Plant Physiol..

[58] Phan HA, Li SF, Parish RW (2012). MYB80, a regulator of tapetal and pollen development, is functionally conserved in crops. Plant Mol. Biol..

[59] Pan X (2020). OsMYB80 regulates anther development and pollen fertility by targeting multiple biological pathways. Plant Cell Physiol..

[60] Liu XQ (2010). The effect of the rice blast resistance gene Pi36 on the expression of disease resistance-related genes. Chin. Sci. Bull..

[61] Mangelsen E (2008). Phylogenetic and comparative gene expression analysis of barley (*Hordeum vulgare*) WRKY transcription factor family reveals putatively retained functions between monocots and dicots. BMC Genom..

[62] Enfissi EMA (2021). New plant breeding techniques and their regulatory implications: An opportunity to advance metabolomics approaches. J. Plant Physiol..

[63] Zhang G (2021). Target chromosome-segment substitution: A way to breeding design in rice. The Crop Journal.

