# Supplementary material for: Comparative genome-wide analysis of WRKY, MADS-box and MYB transcription factor families in Arabidopsis and rice
Source: Sci Rep. 2021 Oct 4;11:19678. doi: 10.1038/s41598-021-99206-y (PMC8490385; doi:10.1038/s41598-021-99206-y)

SUPPLEMENTARY FILE 1

Title: Comparative genome-wide analysis of WRKY, MADS-box and MYB transcription factor families in *Arabidopsis* and rice

Authors: Muhammad-Redha Abdullah-Zawawi, Nur-Farhana Ahmad-Nizammuddin, Nisha Govender, Sarahani Harun, Norfarhan Mohd-Assaad, Zeti-Azura Mohamed-Hussein

Figure 1. Exon-intron structure of WRKY genes from *A. thaliana* and *O. sativa* based on NJ phylogenetic clades. The exons and introns are represented by yellow rectangle and thin grey lines, respectively. Thick blue lines are represented untranslated regions (UTRs).

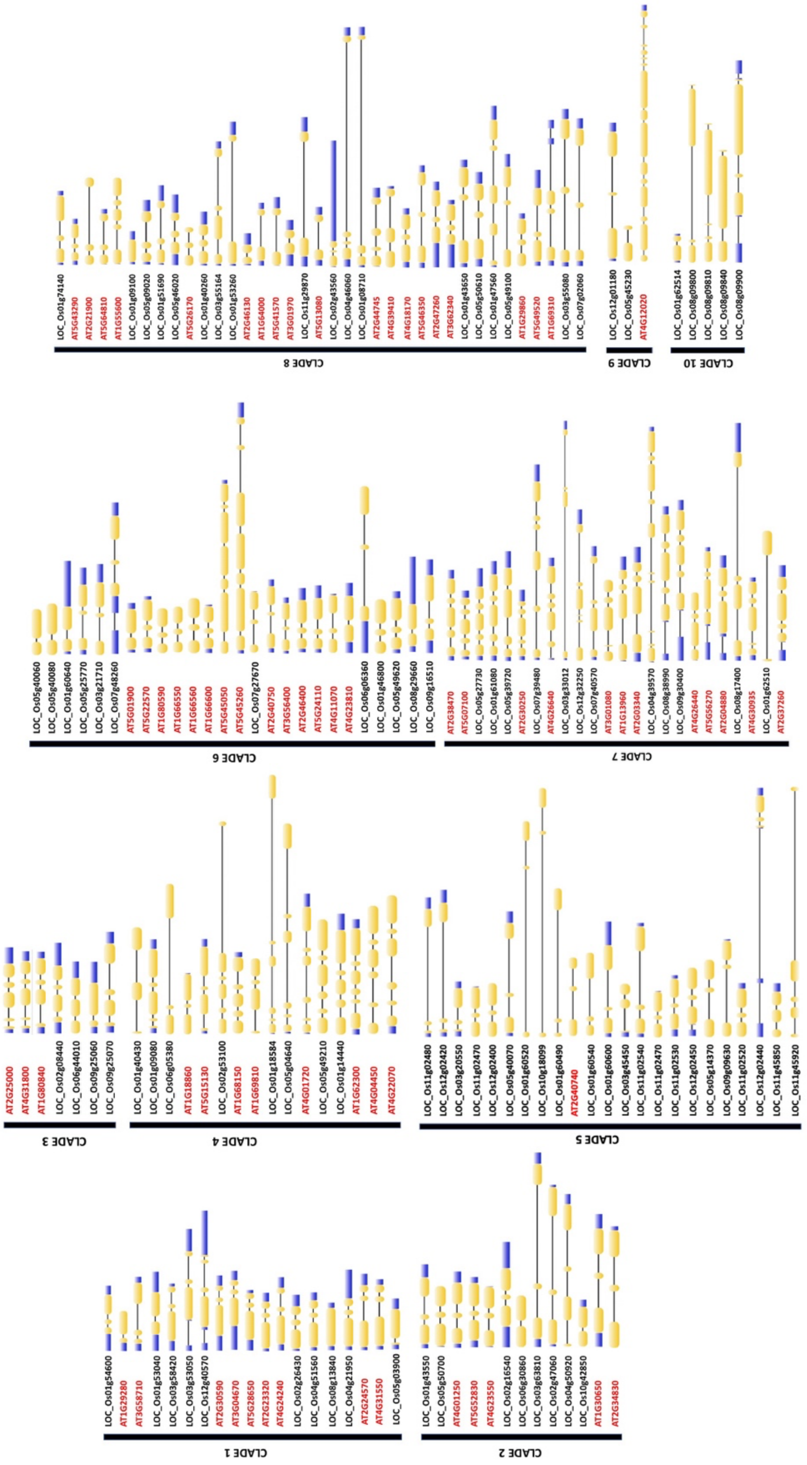

Figure 2. Exon-intron structure of MADS box genes from *A. thaliana* and *O. sativa* based on NJ phylogenetic clades. The exons and introns are represented by yellow rectangle and thin grey lines, respectively. Thick blue lines are represented untranslated regions (UTRs).

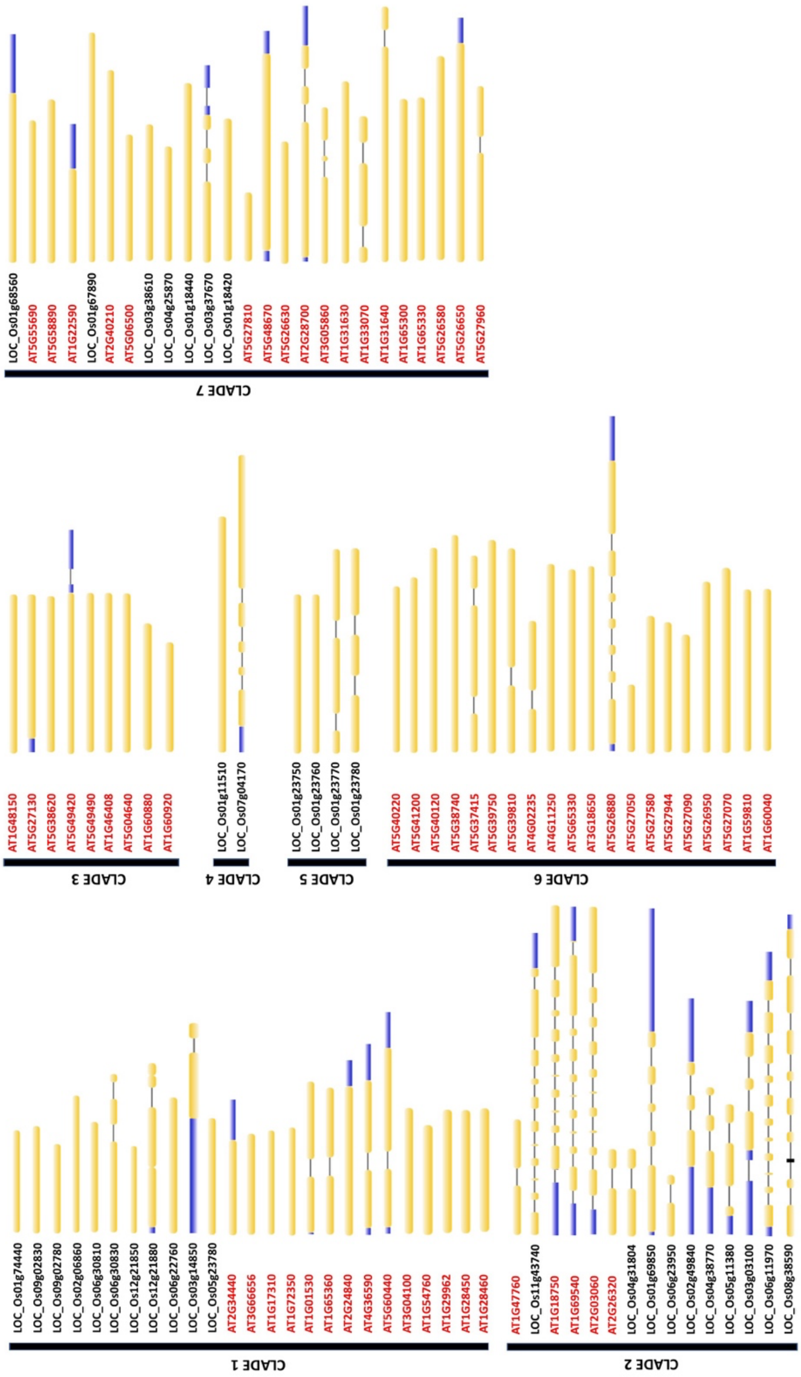

Figure 3. Exon-intron structure of MYB genes from *A. thaliana* and *O. sativa* based on NJ phylogenetic clades. The exons and introns are represented by yellow rectangle and thin grey lines, respectively. Thick blue lines are represented untranslated regions (UTRs).

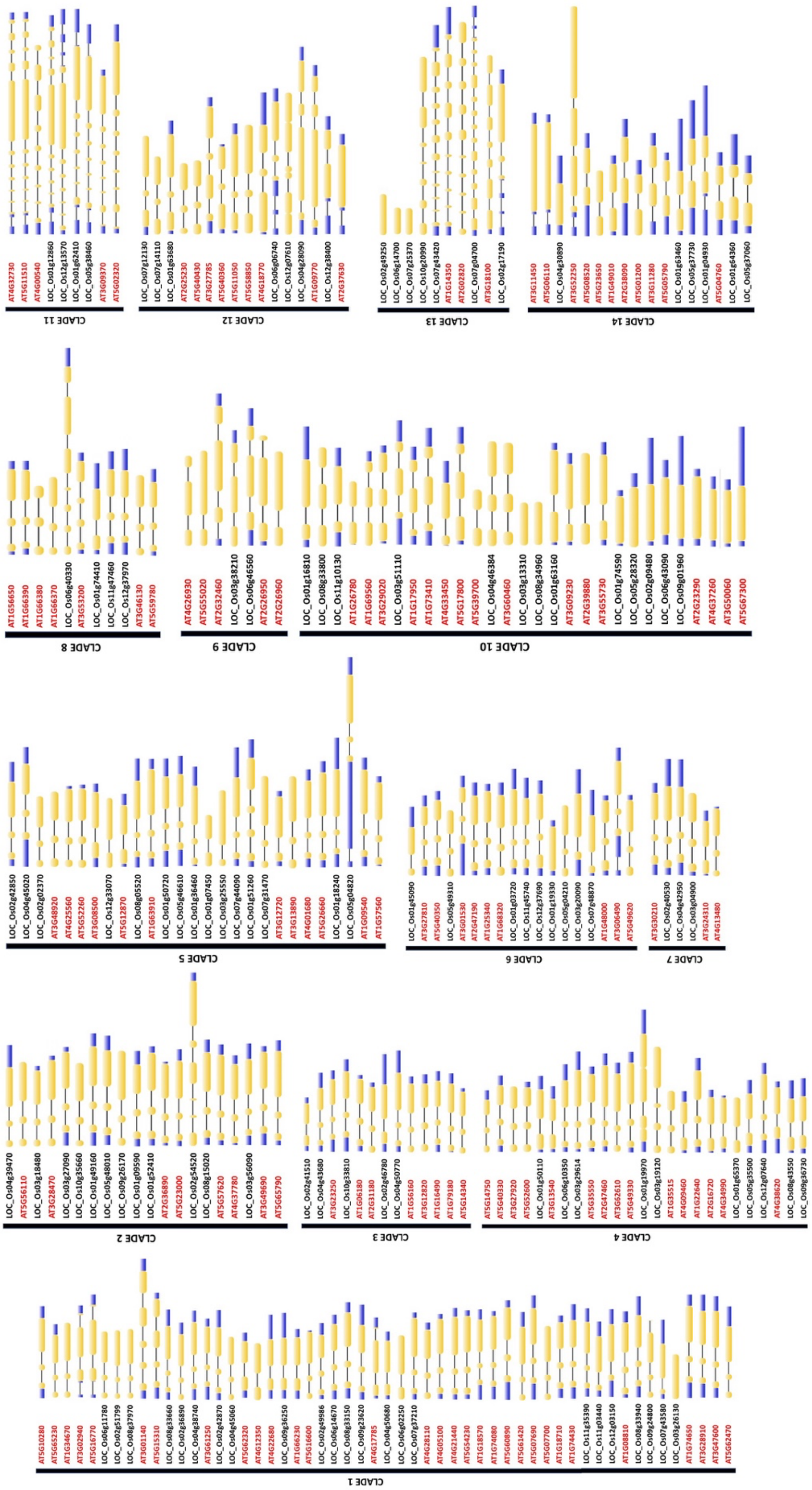

Supplement: Supplementary file 1 — Supplementary Information 1. [file 41598_2021_99206_MOESM1_ESM.pdf]
