# Supplementary material for: Comparative genome-wide analysis of WRKY, MADS-box and MYB transcription factor families in Arabidopsis and rice
Source: Sci Rep. 2021 Oct 4;11:19678. doi: 10.1038/s41598-021-99206-y (PMC8490385; doi:10.1038/s41598-021-99206-y)
Supplement: Supplementary file 2 — Supplementary Information 2. [file 41598_2021_99206_MOESM2_ESM.pdf]

SUPPLEMENTARY FILE 2

Title: Comparative genome-wide analysis of WRKY, MADS-box and MYB transcription factor families in *Arabidopsis* and rice

Authors: Muhammad-Redha Abdullah-Zawawi, Nur-Farhana Ahmad-Nizammuddin, Nisha Govender, Sarahani Harun, Norfarhan Mohd-Assaad, Zeti-Azura Mohamed-Hussein

Table 1. A total of 20 distinct conserved motifs from WRKY, MADS box and MYB proteins of orthogous gene pairs. All motifs were annotated its function by Pfam webserver using motifs consensus sequences. Yellow colored box represents motifs with identified function annotation.

MEME			Pfam		
**Motifs**	**Motifs consensus**	**Length (bp)**	**Family**	**Description**	**E-value**
**WRKY**					
1	ILDDGYRWRKYGQKVVKG NPFPRSYYRCT	29	WRKY	WRKY DNA -binding domain (PF03106)	5.4e-15
2	VZRASDDPSIVITTYEGKHN H	21	WRKY	WRKY DNA -binding domain (PF03106)	1.6e-04
3	GYNWRKYGQKQVKGSEYP RSYYKCTHPNC	29	WRKY	WRKY DNA -binding domain (PF03106)	2.2e-14
4	GCPVRKQ	7	N/A	N/A	N/A
5	VKKKVERSLDGQVTEIVYK GRHNHEPPQP	29	WRKY	WRKY DNA -binding domain (PF03106)	1.2e-05
6	KKGREPRFAFQTRSEVD	17	N/A	N/A	N/A
7	PCEKLMENFEPILTQLQFLS	20	N/A	N/A	N/A
8	WRCPDWNW	8	N/A	N/A	N/A
9	LFAGLGELPECAVVFGRR	18	N/A	N/A	N/A
10	LQEZMERMKEENRRLRKLV EKTLEDYRELZMKF	33	DOG1	Seed dormancy control (PF14144)	1.1e-01
11	FAWFREMECT	10	N/A	N/A	N/A
12	QMQMQM	6	N/A	N/A	N/A
13	YPFFLRDKPRDWPCKKKKI EKFNAQDENKKWQ	32	N/A	N/A	N/A
14	CCGNDDNEDGFFLEPPCFFN RQDKDNMAGLLDGCGFIAN	39	N/A	N/A	N/A

15	NRKARVSVRVRC	12	N/A	N/A	N/A
16	KRSRRSVEKRVVNVPIKEC	19	N/A	N/A	N/A
17	FEWIDQ	6	N/A	N/A	N/A
18	WDFVGHF	7	N/A	N/A	N/A
19	DFPIDF	6	N/A	N/A	N/A
20	PLPVGATAMAST	12	N/A	N/A	N/A
**MADS box**					
1	MGRVKLKIKKLENSSGRQV TYSKRKNGILKKAKELSILC DIDJILLMFSP	50	SRF-TF	SRF-type transcription factor (DNA-binding and dimerisation domain) (PF00319)	7.2e-19
2	CVGEHSCIEEVISKFAQQTP QERAKRKLESLEALKKTFK KLDHDVNIQDF	50	N/A	N/A	N/A
3	RLSCWSDPEKIENIEHJDALE ZSLKESJERIRIHKENFRKNQ LLPJZCA	49	K-box	K-box region (PF01486)	3.0e-03
4	SYPGPF	6	N/A	N/A	N/A
5	HHDQNWPGDEAFGQNWAN QVCGFTPP	26	Connexin4 3	Gap junction alpha-1 protein (Cx43) (PF03508)	6.1e-0.1
6	PNYSSWF	7	N/A	N/A	N/A
7	SMGDPMADNNGGCEQIPH	18	N/A	N/A	N/A
8	IKSIVW	6	N/A	N/A	N/A
9	AQFQND	6	N/A	N/A	N/A
10	PMDFGG	6	N/A	N/A	N/A
11	PHRZLD	6	N/A	N/A	N/A
12	GQTLEM	6	N/A	N/A	N/A
13	DQGCSN	6	N/A	N/A	N/A
14	PGDSAF	6	N/A	N/A	N/A
15	VAIVZA	6	N/A	N/A	N/A

16	LFDNDA	6	N/A	N/A	N/A
17	CLGLQQLG	8	N/A	N/A	N/A
18	NDKPTI	6	N/A	N/A	N/A
19	EEDQEK	6	N/A	N/A	N/A
20	GGGAEA	6	N/A	N/A	N/A
**MYB**					
1	RCGKSCRLRWINYLRPDJKR GEFTEEEEE	29	Myb_DNA -bind_6	Myb-like DNA-binding domain (PF13921)	1.9e-06
2	LIIRLHAILGNRWSLIAARLP GRTDNEIKNYWNSTJKKRL	40	Myb_DNA -binding	Myb-like DNA-binding domain (PF00249)	2.1e-11
3	KRGPWSPEEDEKLIAYVKK YGPGNWSLIP	29	Myb_DNA -binding	Myb-like DNA-binding domain (PF00249)	1.4e-10
4	WRAIAPGVGRTPFQCLERY ZKLLDAACLKDEWYEEEDD PRKLRPGEFGPN	50	Myb_DNA -bind_6	Myb-like DNA-binding domain (PF13921)	4.1e-05
5	RMGIDPVTHKPF	12	N/A	N/A	N/A
6	MGRHPCCEKEG	11	N/A	N/A	N/A
7	DLDEDCKEMLEGARAWLA NTKGKKWKRKRREKQLEE ERRLR	41	N/A	N/A	N/A
8	IMMEAENLARLRESQTPLL GGENPELHPSDFSGVTPRKK EIQTPNPMATP	50	N/A	N/A	N/A
9	NKIAQRQDAPAAILQANKL NDPEVVRKRSKLMLPPPQIS DHELEEIAKMG	50	LysM	LysM domain (PF01476)	8.1e-02
10	HHHHQHHHNH	10	N/A	N/A	N/A
11	HLMAEIATTLNPPQVAHLA EAALGCFKDEMLHLLTKKR	38	N/A	N/A	N/A

12	DFDEDELKEADKMJEEEGK FLCVAMGHENESLEDFVEA HDACQEDLMFFP	50	Myb_Cef	pre-mRNA splicing factor component (PF11831)	5.0e-04
13	TPRIGLTPSRDGSSFGLTPKG TPFRDELRINEEMDMQ	37	Myb_Cef	pre-mRNA splicing factor component (PF11831)	4.2e-06
14	GFYDTVGEDRPAEQVKFPT TIEELEGKRRVDIEAQLRKQ D	40	N/A	N/A	N/A
15	YSQTPRQGMTPLRTPQRTP AGKGD	24	N/A	N/A	N/A
16	TELECFKELKKQEZMAASF RKKNLQEEVNKQKELERKL QSRYG	43	N/A	N/A	N/A
17	NRACQKFJRGKLIEIEAKIEZ NEDHKDRVKCLKDFQASCK KIFGKILCQ	49	DUF4294	Domain of unknown function (DUF4294) (PF14127)	3.3e-02
18	EYREKLVGLRRDAEAKEQK LAEQWAAKHIRLAKFLEQQ AGCR	42	N/A	N/A	N/A
19	PQPKNEYQIV	10	N/A	N/A	N/A
20	HHHHHP	6	N/A	N/A	N/A
